# A Comprehensive Review of the Triangular Relationship Among Diet, Gut Microbiota, and Aging

**DOI:** 10.3390/ijms26188785

**Published:** 2025-09-09

**Authors:** Chapa Ramasinghe, Matteo Bordiga, Baojun Xu

**Affiliations:** 1 Food Science and Technology Program, Department of Life Sciences, Beijing Normal-Hong Kong Baptist University, Zhuhai 519087, China; chapamramasinghe@gmail.com; 2 Department of Food Science and Technology, Faculty of Agriculture, University of Peradeniya, Peradeniya 20400, Sri Lanka; 3 Department of Pharmaceutical Sciences, Università del Piemonte Orientale, Largo Donegani, 228100 Novara, Italy; matteo.bordiga@uniupo.it

**Keywords:** gut microbiota, healthy aging, diet, dysbiosis, microbial metabolites

## Abstract

Aging is a complex biological process influenced by internal and external factors, with diet and gut microbiota emerging as pivotal, interconnected modulators. This review explores their triangular relationship, emphasizing how they dynamically interact to shape health across the lifespan. Aging involves notable shifts in gut microbiota, including reduced diversity, increased pro-inflammatory taxa, and impaired production of key metabolites, like short-chain fatty acids. These changes contribute to systemic inflammation, immune-senescence, and age-related conditions, such as cognitive decline and metabolic disorders. Diet, particularly Mediterranean and plant-based patterns, plays a critical role in modulating gut microbiota by enhancing beneficial microbes and their metabolic functions. In contrast, Western-style diets rich in saturated fats and processed foods promote dysbiosis and accelerate aging. The review synthesizes evidence from human studies, animal models, and interventions to show how microbiota mediates diet-driven effects on aging. It also explores the role of specific nutrients, fiber, omega-3 fatty acids, and polyphenols in influencing microbial and host aging biology. Emerging therapies, including probiotics, prebiotics, and precision nutrition, show promise for promoting healthy aging by restoring microbial balance. However, gaps remain, including the need for long-term, age-specific studies, standardized microbiome protocols, and integrated omics approaches to support targeted longevity strategies.

## 1. Introduction

The human gut microbiome has become a great interest for the majority of research in the past few decades. Previously being overlooked in favor of studying specific harmful or beneficial microbes, in later years, research on the overall structure of the gut microbiome has grown rapidly [1]. Scientists who have different research interests have caused a considerable increment in reports, which show how the gut microbiota changes depending on a person’s age, health, diet, and the place he or she lives [1]. The variety of the gut microbiota and its number change throughout life from birth to death. It is well known that the microbiota composition rapidly changes during the early stages of life; then, changes occur at a slower rate until around age 40, and then throughout the latter stages. It becomes somewhat more stable compared to the early stages of life. This stability again is lost when the person gets older, as microbes living in the body also age, depending on multiple factors (disease, medication, and stress) [2].

Gut microbes could send different signals to the brain. These signals affect behavior, mainly thinking and memory, through a pathway called the “gut–brain axis”. This signaling mechanism is performed using nerve signals, hormones, and the immune system. Together, the microbiome can provide people with a new and exciting way to understand how both the body and the mind change with time, which is known as aging [3].

Diet has the biggest effect on the immune system–microbiota working together. Due to this effect, many people are very interested in personalized nutrition or precision nutrition to help treat and prevent different diseases and illnesses [4].

In this context, the present review focuses on the triangular relationship among diet, gut microbiota, and aging. Specifically, this review aims to synthesize the current understanding of the diet–gut microbiota–aging axis, offering insights into how dietary patterns and specific nutrients can shape the composition and function of the gut microbiota and thereby influence the biological processes associated with aging. By examining the mechanistic pathways that link diet and microbiota to age-related physiological changes, as well as the therapeutic potential of modulating this triad, we seek to support the development of targeted strategies for promoting healthy aging and reducing the risk of age-associated diseases.

## 2. Methodology

A comprehensive literature search was conducted across multiple scientific databases, including PubMed, Scopus, Web of Science, and Google Scholar, to identify relevant studies examining the relationship between diet, gut microbiota, and aging. The search strategy applied a combination of keywords and Boolean operators. The keywords included “diet and gut microbiota”, “Mediterranean diet and microbiome”, “Western diet and gut health”, “plant-based diet and aging”, “dietary patterns and microbiota composition”, “microbiota and longevity”, “aging and gut dysbiosis”, and “microbiota-derived metabolites and health”. In addition, specific terms such as “short-chain fatty acids” (SCFAs), “tryptophan metabolites”, “oxidative stress”, “inflammation”, “cognitive decline”, and “immune regulation” were incorporated to capture mechanistic insights.

The inclusion criteria were (1) studies published in peer-reviewed journals between 2000 and 2025, (2) articles available in English, (3) studies focusing on the interaction between dietary patterns or nutrients, gut microbiota composition, and markers of biological aging, and (4) original research articles, reviews, and meta-analyses. Exclusion criteria included case reports, short communications, conference abstracts, and studies not directly linking diet, microbiota, and aging outcomes.

The search process followed a stepwise approach as follows: (i) initial screening of titles and abstracts to remove irrelevant articles, (ii) full-text review of potentially eligible studies, and (iii) removal of duplicate records. Additional manual searching for references from key publications was also performed to broaden coverage. Studies were further filtered based on methodological quality and clarity of reported outcomes. This strategy ensured that the review incorporated high-quality, relevant, and up-to-date evidence to critically evaluate the role of diet and gut microbiota in shaping the aging process.

## 3. Gut Microbiome—Overview

### 3.1. Complexity and Composition of Gut Microbiota

The human gut, with its 200–300 m^2^ of surface area, is home to around 30–40 trillion different microbes (symbionts including 50 bacterial groups and 100–1000 species), together called the “gut microbiota”. This is roughly comparable in number to human cells, giving an approximate 1:1 ratio. These microbes outnumber the body’s own cells by about ten to one. The combined genetic material of these microbes is called the “microbiome”, and it is about 150 times larger than the human genome. In each person, around 150–170 main bacterial species thrive in the gut’s warm, nutrient-rich environment, where they carry out important protective, metabolic, and structural roles [5].

### 3.2. Dominant Bacterial Groups

**Figure 1:**
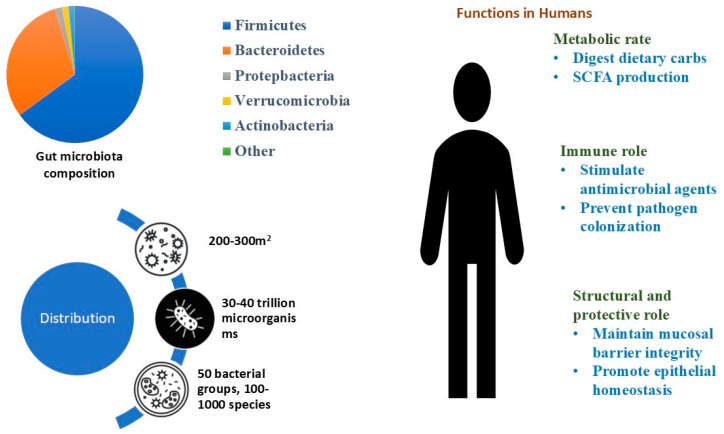
Composition, distribution, and functions of gut microbiota.

The human gut microbiota is made up of various microorganisms, including viruses, bacteria, archaea, parasites, and eukaryotic microbes. The microenvironment of the gut mainly supports the growth of bacteria that belong to seven dominant groups: *Bacteroidetes*, Proteobacteria, Firmicutes, Cyanobacteria, Verrucomicrobia, Actinobacteria, and Fusobacteria (Figure 1). When analyzing these, Firmicutes and *Bacteroidetes* together account for over 90% of the prevailing bacterial population. Many of the bacterial species that belong to the phylum *Bacteroidetes* are categorized under the genera *Prevotella* and *Bacteroides*. In the Firmicutes group, key species include *Clostridium* clusters IV and XIVa, which are particularly from the genera *Ruminococcus*, *Clostridium*, and *Eubacterium*, the genera that are predominant in the gut [6].

### 3.3. Early-Life Microbiota Development

The human microbiome experiences several crucial changes throughout life. From birth, there is an accelerated growth of different microbial species that help to form the infant’s microbiome, leading to an initial development of the infant’s microbiome, which varies depending on the mode of delivery and whether the infant is fed formula, milk, or breast milk. Around the age of three, almost all the gut microbiota begin to stay more stable, fully developed, and strong. This stability continues into adulthood, with minor changes caused by daily cycles and lifestyle habits like diet, physical activity, smoking, the use of antibiotics, and place of residence [7].

Children who were only fed breast milk tend to have a higher number of beneficial bacteria, especially *Bifidobacteria*, than children who received formula milk. When milk is replaced with solid food, the gut microbiome undergoes significant changes. After this shift, Firmicutes and *Bacteroidetes* become the main types of bacteria and remain dominant throughout childhood [6].

The composition of the gut microbiome gradually grows over time, along with the ability to digest complex carbohydrates and foreign compounds, like xenobiotics, to form vitamins. By the age of three, a child’s microbiota is similar to that of an adult, although some groups of microbes only fully develop during adolescence [6].

### 3.4. Functional Roles of Microbiota in Adults

Gut microbiota has several important functions that contribute to the wellness and health of an individual. The gut microbiome, rather than the human genome, plays a crucial role in the metabolism of dietary carbohydrates [8]. Bacteria in the gut assist in the development of the immune system, protect against pathogenic microbes, and play an important role in xenobiotic metabolism, aiding in the digestion of carbohydrates. During the digestion of carbohydrates, short-chain fatty acids (SCFAs) such as acetate, propionate, and butyrate are produced. The amount of carbohydrate intake, along with the ability of the microbes to inhabit the intestine, determines the type and amount of fatty acid chain synthesized [9].

Through collaboration with species specialized in oligosaccharide fermentation (e.g., *Bifidobacteria*), Firmicutes and *Bacteroidetes* produce SCFAs from indigestible carbohydrates that escape digestion in the small intestine. SCFAs constitute approximately two-thirds of the colonic anion concentration (70–130 mmol/L) [8].

Some of the most important roles of these microbes are to help maintain the integrity of the mucosal barrier, to provide nutrients, such as vitamins, or to protect against pathogens. In addition, the interaction between commensal microbiota and the mucosal immune system is crucial for proper immune function [10]. They contribute to epithelial homeostasis, promote wound healing, and regulate mucus production and glycosylation. Through interactions with immune receptors, they stimulate the production of antimicrobial peptides, secretory IgA, and anti-inflammatory mediators while also preventing pathogen colonization. Disruptions in this microbial ecosystem due to antibiotics or poor diet can impair these functions, increasing susceptibility to infections, inflammation, and metabolic disorders [10].

### 3.5. Dysbiosis and Its Link to Diseases

A healthy host–microorganism balance must be respected in order to optimally perform metabolic and immune functions and prevent disease development [11]. Various perinatal determinants, such as cesarean section delivery, type of feeding, antibiotic treatment, gestational age, or environment, can affect the pattern of bacterial colonization and result in dysbiosis. The alteration of the early bacterial gut pattern can persist over several months and may have long-lasting functional effects with an impact on disease risk later in life. In elderly individuals, reduced diversity and loss of beneficial taxa weaken intestinal barrier function and promote low-grade systemic inflammation. This immune dysregulation contributes to conditions such as type 2 diabetes, cardiovascular disease, and frailty. In parallel, microbial metabolites, including short-chain fatty acids and tryptophan derivatives, interact with the neuroendocrine system through the gut–brain axis, influencing cognitive decline and mood disorders. These diseases, by sustaining chronic inflammation and oxidative stress, accelerate biological aging and weaken resilience against further insults. Neurodegenerative changes, vascular damage, and metabolic dysfunction amplify functional decline, thereby linking dysbiosis-driven disorders directly to unhealthy aging. Dysbiosis is, therefore, not only a feature of aging but also a driver of its comorbidities. Hence, the gut microbiota interacts extensively with the host, and there are accumulating data linking many human diseases—such as inflammatory bowel disease, obesity, diabetes, asthma, and allergies—with dysbiosis, that is, an alteration in its composition [12].

### 3.6. Changes in Microbiota Composition

The composition of the human gut microbiota varies along the GI tract due to variations in pH, O_2_ tension, digesta flow rates, substrate availability, and host secretions. Due to infant transitions and age, changes may occur within the same individual. The small intestine provides a more challenging environment for microbial colonizers, given the fairly short transit times (3–5 h) and the high bile concentrations. In contrast, the large intestine, characterized by slow flow rates and neutral to mildly acidic pH, harbors by far the largest microbial community [11]. A shift in diet composition can have an impact on this ratio as gut microbiota responds quickly (within 24 h) to changes in diet in both people and mice [13].

## 4. Diet as a Modulator of the Gut Microbiota

Dietary habits are a major factor influencing intestinal microbial composition [14]. The way that diet interacts with the gut microbiome and overall metabolism is not straightforward; it is a web of influences moving in different directions. This complexity makes it difficult to apply a standardized model of dietary guidelines in practice. However, recent scientific research suggests that what people consume plays a significant role in shaping or modulating the gut microbiome. Changes that happen in the gut microbial composition affected by dietary patterns have been tied to many illnesses and their relevant metabolic issues. Among dietary components, dietary fiber has drawn particular interest for its positive potential to influence human gut metabolism by modulating the gut bacteria [15].

Long-term dietary interventions appear to be more effective in reshaping the gut microbiota. While instant, short-term dietary changes can still affect microbial balance, the adult gut tends to return to its baseline due to its ability of resilience. This resilience is closely tied to long-term consistent dietary patterns, which offer a steady supply of nutrients and gradually shape the gut microbial composition [16].

As an example, the dietary carbohydrate intake may help to explain the wide range of differences seen in gut microbiota profiles between individuals. High-fiber diets, in particular, are often linked with greater microbial diversity. While high-fiber diets positively affect the gut microbial composition, in contrast, high-fat diets tend to disrupt gut microbial balance, commonly leading to a drop in beneficial bacteria, such as *Roseburia* [14].

As explained above, since long-term dietary interventions are believed to strongly influence the gut microbial composition, a person’s ability to adopt and maintain a dietary pattern should be considered when considering the diet–microbe relationship. Long-term modifications to diets can promote the growth of new microbial species and support the expansion of beneficial ones, ultimately enhancing microbial diversity and richness. Over time, this may nurture a more stable and balanced gut ecosystem, with potential benefits for overall host health [17].

## 5. Impact of Dietary Patterns on Gut Microbiota

The concept of dietary patterns has recently gained more attention as a broader framework for understanding how eating habits influence chronic disease risk. Rather than the identification of individual food intakes, this acts as a universal approach that considers the combined impact of the overall diet as a cluster [18]. Diet is one of the strongest modulators of gut microbiota composition, with even short-term dietary changes leading to rapid shifts in microbial communities [19].

The interaction between diet and the gut microbiota is bidirectional. While gut microbes act on the digested nutrients that reach the intestine, on the other hand, nutrient intake through diet plays a powerful role in shaping the microbial community and the composition of the gut microbiome. The metabolic activity of these microbes depends heavily on the supply of non-digestible carbohydrates and proteins. Studies in both animals and humans have shown that any kind of dietary change can modify the composition of gut microbiota. In healthy individuals, a well-balanced diet may ensure the development of a stable and balanced microbial ecosystem, where bacterial populations coexist in a controlled and mutual balance [20].

Furthermore, the composition of the gut microbiota can be substantially improved by the intake of probiotics, the live microorganisms. These probiotics are metabolically active in the colon, and they may temporarily colonize the gut and influence microbiota metabolism. Prebiotics, typically non-digestible fibers, also play a key role by stimulating the growth of beneficial bacterial species [21].

**Figure 2:**
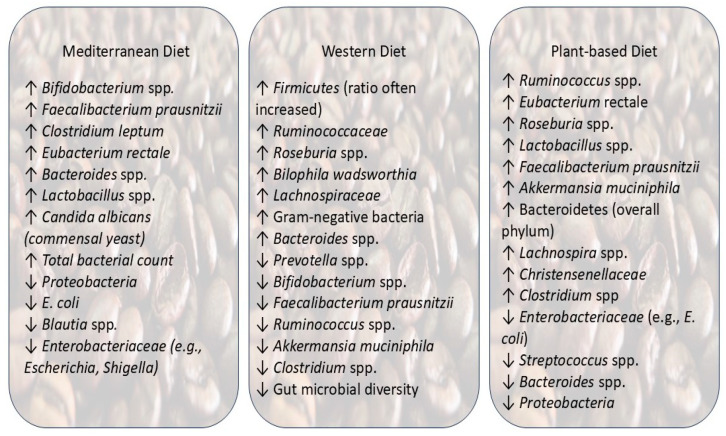
Compositional changes in gut microbiota due to intervention of different dietary patterns (The upward arrow (↑) indicates increment, while the downward arrow (↓) indicates decrease).

The three main dietary patterns in the world are the Mediterranean diet, the Western diet, and plant-based diets. These three types can affect the gut microbiome in different ways (Table 1) (Figure 2).

### 5.1. Mediterranean Diet

The Mediterranean diet (MD), acknowledged by UNESCO (United Nations Educational, Scientific, and Cultural Organization) as part of the world’s intangible cultural heritage, is widely recognized for its nutritional benefits. It emphasizes high consumption of fruits, vegetables, legumes, nuts, and minimally processed cereals, includes moderate amounts of fish, limits the intake of saturated fats, red meat, and dairy, and allows for regular but moderate alcohol consumption, typically in the form of red wine during meals [22]. The main source of dietary lipid in the MD is olive oil, and the diet requires an adequate amount of water each day [18]. Other than recommending different dietary components, the MD emphasizes physical activity to support both physical and mental well-being. A great advantage of this diet is its dependence on traditional and locally found food products, aligning food choices with seasonal harvests and promoting a diverse intake of food products [18].

Beyond its widely recognized nutritional value, greater adherence to the Mediterranean diet has been associated with a notable decline in both mortality and morbidity indicators [23]. Particularly, the MD has also been linked to an increment in beneficial gut microbiota composition, including a decline in proteobacterial levels and enhanced production of short-chain fatty acids (SCFAs) [32]. The elevated fecal SCFA levels observed normally reflect the increased microbial fermentation of indigestible carbohydrates that reach the colon [22].

The favorable shifts in gut microbiota linked to the MD are mainly due to dietary components such as dietary fiber, extra virgin olive oil, and polyunsaturated fats (PUFAs), particularly omega-3 fatty acids. These components support the growth of SCFA-producing bacteria, like *Clostridium leptum* and *Eubacterium rectale*, as well as other beneficial taxa, including *Bifidobacteria*, *Bacteroides*, and *Faecalibacterium prausnitzii*. In contrast, a notable decline in certain Firmicutes, particularly *Blautia* species, has also been observed [33].

One special observation in MD adherence is its potential to reduce harmful bacteria. Studies prove that high adherence to the MD was associated with lower counts of *E. coli*, an indicator pathogenic bacterium. Additionally, the MD has improved the *Bifidobacteria*/*E. coli* ratio, an indicator of gut microbial balance and overall health of a person. Similarly, sustained intake of polysaccharide-rich diets has been linked to declines in *Enterobacteriaceae*, such as *Shigella* and *Escherichia*, and dietary antioxidants can retard the growth of *E. coli* strains [22]. In line with this, another study, utilizing the MedDietScore, a validated index measuring adherence to the Mediterranean diet, observed that individuals with higher MedDietScores exhibited lower levels of *E. coli*, increased total bacterial counts, improved *Bifidobacteria*/*E. coli* ratios, and had a greater prevalence of *Candida albicans* and short-chain fatty acids (SCFAs) [23].

Despite these well-documented advantages, older adults worldwide have limited adaptation to the MD. One of the major challenges in elderly healthcare is the prevalence of restricted diets, particularly in long-term care settings, which is linked to a reduction in gut microbiome diversity. In a 6-month dietary intervention study, elderly participants received a daily supplement of 20 g of five prebiotic compounds. Even though this supplementation showed microbial shifts, no considerable changes in overall diversity or statistically strong reductions in inflammatory markers were observed. This suggests that even though prebiotics, like therapeutic supplements, are given without a broader adoption of the MD, measurable improvements may not be produced [32].

### 5.2. Western Diet

Over the last hundred years, industrial agriculture and modern food processing have significantly changed the way people eat by creating a critical shift toward diets rich in saturated fats, refined sugars, salt, and artificial sweeteners while reducing the intake of fiber-rich, plant-based carbohydrates [34]. The Western diet, often defined by these characteristics, has become a major factor influencing gut microbiota composition and function.

This Western dietary pattern, typically high in saturated fats and simple sugars, has been strongly linked to gut dysbiosis, a disruption in the normal microbial balance. Western diets modify the diversity, structure, and metabolic capabilities of the gut microbiota. They diminish the presence of beneficial saccharolytic bacteria that ferment fiber while fostering the growth of bile-tolerant and potentially harmful microorganisms, such as *Bilophila wadsworthia*. These alterations may occur within just 1–2 days of adopting a Western diet, and some shifts may persist even after reverting to a healthier dietary pattern [35]. More importantly, high-fat diets, which are one of the main components of a Western diet, can enhance the ratio of Firmicutes to *Bacteroidetes*, although this outcome is not always consistent across individuals.

While being consistent with previous observations, a study on Syrian hamsters revealed that a Western diet marked by high fat and high fructose could significantly induce rapid shifts in microbial communities just within a week. The Western diet led to an elevated ratio of Firmicutes/*Bacteroidetes* bacteria and a marked drop in beneficial genera, like *Prevotella*. Simultaneously, an expansion in microbial taxa, such as *Ruminococcaceae* and *Roseburia*, was observed, reflecting the fact that alterations have taken place in microbial metabolic output, including the production of short-chain fatty acids and branched-chain amino acids [28]. Moreover, commonly used emulsifiers in processed foods, such as carboxymethylcellulose (CMC), have been shown to negatively impact the gut microbiota by reducing the populations of beneficial microbes, like *Faecalibacterium prausnitzii* and *Ruminococcus* spp., while increasing *Roseburia* sp. and *Lachnospiraceae*, which were proven through human trials. Even though *Roseburia* is usually considered beneficial, its role is not absolute. Abundance can vary depending on diet quality and interactions with other microbes. In low-fiber, high-fat contexts, its increase does not necessarily mean positive outcomes because metabolite balance and microbial interactions are altered. Furthermore, although fecal lipopolysaccharide (LPS) levels were not significantly changed, there is a possibility of compromising the gut barrier integrity and disruption of host–microbiota interactions [36]. Ultra-processed foods, which are a staple of the Western diet, have also been linked to microbiota imbalances favoring pro-inflammatory bacterial species.

These negative alterations in gut microbiota composition are further enhanced by other Western diet components such as refined sugars, acellular nutrients, and food additives. Such components can modify the microbial composition, reduce the gut barrier integrity, and impair immune function. Furthermore, some studies show that these diet-mediated microbial changes may be transmitted across generations through epigenetic mechanisms, implying the negative effect of long-term poor dietary choices and the requirement of food regulation that prioritizes microbial health [37].

Both animal and human studies have demonstrated the fact that the consumption of red and processed meat, a typical dietary component of the Western diet, can significantly influence the gut microbiome composition. Individuals with high intake of these foods have a gut microbial composition predominated by *Bacteroides*-rich microbiota rather than a *Prevotella*-rich one. This microbial profile is noteworthy because *Bacteroides* species are more efficient at converting dietary *L*-carnitine, prevalent in red meat, into trimethylamine (TMA), a compound subsequently oxidized in the liver to form trimethylamine-N-oxide (TMAO), a metabolite strongly associated with atherosclerosis and adverse cardiovascular outcomes [38]. Additionally, the Western diet, which is high in saturated fatty acids (SFAs), has been shown to promote the proportion of Gram-negative bacteria, potentially contributing to further microbial imbalance and inflammation [39]. In addition to altering microbiota composition, the Western diet disrupts microbial function by modifying the nutrient landscape available to gut microbes. These changes can impair microbial signaling pathways and weaken gut barrier integrity. While a balanced diet supports microbial homeostasis and barrier function, the pro-inflammatory nature of Western dietary patterns contributes to compromised epithelial defenses and dysregulated host–microbe communication, establishing a self-reinforcing cycle of gut dysbiosis [34].

### 5.3. Plant-Based Diets (Vegetarian/Vegan)

A plant-based diet is generally characterized by its high intake of plant-based foods while limiting the consumption of animal-based products. The diet usually includes whole grains, fruits, vegetables, legumes, and nuts, which have been associated with reduced risk of several chronic conditions [40]. The specific composition of such diets appears to play a pivotal role in shaping the gut microbiota.

Both vegetarian and vegan diets are associated with a distinct gut microbial profile characterized by greater overall diversity and increased abundance of several taxa involved in the metabolism of plant polysaccharides. These vegetarian and vegan diets are often characterized by their potential to increase levels of *Ruminococcus*, *Eubacterium rectale*, and *Roseburia*, while shifting the *Bacteroidetes* to Firmicutes ratio in a significant manner. These changes appear to stem from high intake of dietary fiber and polyphenols, along with limited consumption of animal-based nutrients. Collectively, these adaptations foster an intestinal environment better equipped to ferment complex plant substrates and support gut health [41].

Microbial diversity has been positively associated with increased variety in plant-based food intake. Consumption of a broad range of plant-based products offers a higher range of substrates that support the proliferation of multiple microbial taxa [17]. As an example, consumption of dietary fiber and resistant starch has been linked to a significant increment in microbial diversity and selectively promotes the growth of species like *Lactobacillus*, *Ruminococcus*, and *E. rectale*, which ferment these substrates in the colon [42].

On the other hand, going beyond the shaping of microbial composition, plant-based diets lead to meaningful functional adaptations within the gut ecosystem. Continuous intake of a vegetarian diet has been linked to a diminished microbial capacity for carnitine metabolism, a compound abundant in red meat that can be converted to pro-atherogenic metabolites, such as trimethylamine-N-oxide. Simultaneously, an enhanced expression of microbial genes related to nitrogen assimilation was observed, reflecting a lower intake of animal-derived amino acids. These findings illustrate a metabolic reprogramming of the gut ecosystem in response to plant-based nutrient inputs [30].

Consistently, individuals adhering to vegetarian diets exhibit relative abundances of *Clostridium* species and *Bilophila wadsworthia* compared to omnivores, suggesting a gut microbiota composition that is less dependent on animal-based products [43]. A study reported the same kind of result, indicating that the people who adhere to vegetarian diets show particularly elevated levels of *Bacteroidetes*-related taxa when compared to omnivores. These observations suggest that sustained plant-based eating supports a more complex and diverse microbial ecosystem [44].

Vegans, in particular, display a distinct gut microbial profile. One study reported that exclusive consumption of plant-based foods was associated with elevated abundance of *Bacteroidetes* and higher levels of *Lachnospira*, a genus specialized in fiber fermentation. Concurrently, reduced levels of *Enterobacteriaceae* and *Streptococcus* were observed, suggesting a microbial community that is less prone to pro-inflammatory activity [45]. Another study reported an elevated abundance of *Akkermansia muciniphila*, a mucin-degrading bacterium known for reinforcing gut barrier integrity and exerting anti-inflammatory effects, adding further support to the view that vegan dietary patterns promote intestinal homeostasis [46].

However, plant-based diets do not uniformly elevate all beneficial microbial taxa. In one study, individuals adhering to a vegan diet exhibited significantly lower levels of *Bifidobacterium* and *Bacteroides* compared to omnivores, potentially reflecting the reduced intake of dietary fats and proteins. This suggests a distinctive microbial restructuring, where certain populations decline while others, particularly those adept at utilizing complex carbohydrates, proliferate in response to the prevailing nutrient landscape [31]. Additionally, adherence to a vegan diet has been associated with a lower stool pH, a shift that coincided with decreased populations of *Escherichia coli* and *Enterobacteriaceae*. This more acidic gut environment may contribute to the suppression of potentially pathogenic microbes, supporting a microbial ecosystem less conducive to inflammation and disease [47].

Collectively, these findings underscore that vegetarian and vegan diets influence not just the composition of the gut microbiota but also its functional and ecological dynamics. By enhancing microbial diversity, shifting nutrient-processing pathways, and altering gut pH and overall richness, plant-based eating patterns appear to cultivate a microbial ecosystem finely tuned for the fermentation of complex carbohydrates and the support of host health.

## 6. Role of Specific Nutrients in Microbiota–Aging Interactions

**Figure 3:**
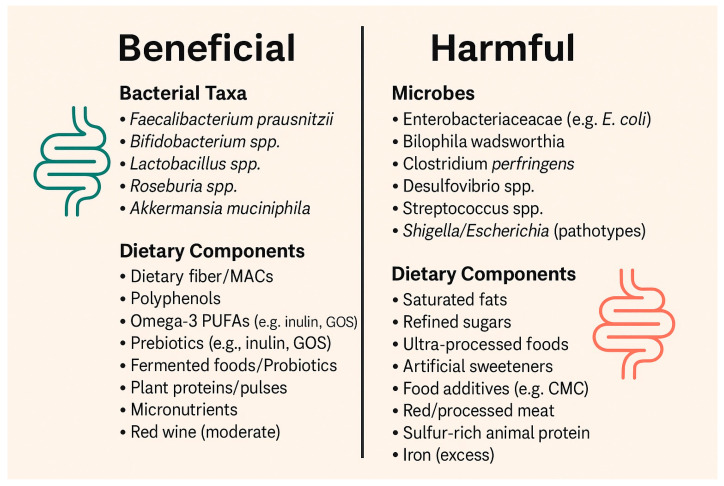
Beneficial and harmful gut microbiota, along with dietary components.

Specific nutrients, such as carbohydrates, fiber, proteins, fat, vitamins, minerals, polyphenols, and therapeutic compounds, like prebiotics, can influence gut microbiota composition along with probiotics by promoting beneficial taxa that produce anti-inflammatory metabolites, like SCFAs. In return, these microbial changes modulate aging-related processes by enhancing gut barrier integrity, reducing systemic inflammation, and supporting metabolic and cognitive health (Table 2) (Figure 3).

### 6.1. Carbohydrates and Fiber

Shaping gut microbial composition and metabolic activity is one of the major roles played by carbohydrates, especially non-digestible dietary fiber. While digestible carbohydrates provide energy for the host, other fibers, including oligosaccharides and resistant starches, are fermented to short-chain fatty acids (mainly butyrate) by gut microbes. Through this fermentation, microbial diversity is promoted, and the proliferation of taxa adapted to plant-derived substrates is enhanced while establishing a metabolically active and resilient gut ecosystem [13].

The beneficial impacts of dietary fiber on gut microbiota have been discussed in several studies. Such a study reported that supplementation with microbiota-accessible carbohydrates increased microbial richness and promoted the growth of *Bacteroides*, *Prevotella*, *Bifidobacterium*, and *Faecalibacterium*, species known for fermenting prebiotics into SCFAs, like acetate and butyrate. These shifts were involved in improved gut barrier integrity, thicker mucus layers, and better cognitive performance [63]. Another study observed that greater fiber intake by older adults led to increased abundances of *Bifidobacterium*, *Lactobacillus*, and *Akkermansia* while increasing SCFA levels. This reinforced the idea that fiber sustains fermentative microbes and supports gut health with age [64].

Across diverse populations, microbial responsiveness to fiber is consistent. A study that utilized 21 short-term dietary fiber interventions observed consistent shifts in gut bacterial communities, even though the participants showed high individual variation, mainly in genera such as *Bifidobacterium*, *Lactobacillus*, *Ruminococcus*, and *Prevotella* [65]. Another study reported that higher plant-derived fiber intake by older adults who are at risk of frailty showed elevated levels of *Faecalibacterium prausnitzii* and *Bifidobacterium*. The SCFAs that were produced by these taxa contributed to the improvement of muscle strength and preservation through enhanced mitochondrial function and anabolic muscle signaling [66].

The host’s metabolic activities and immune health are linked to the production of SCFAs. In a study, mice were fed fiber-enriched high-fat diets. This diet elevated butyrate and acetate levels, and enrichment of microbial diversity was observed. Further, enhancement of intestinal epithelial barrier function was also observed while fueling colonocytes and suppressing inflammation. As a result, *Akkermansia* and *Bifidobacterium* species were also found in elevated levels [67]. Further, another study supplied microbiota-accessible carbohydrates to a *Clostridium difficile* infection (CDI) model, which resulted in elevated SCFA production while suppressing pathogen growth and reducing microbial burden. Even though animal studies help to understand how carbohydrates affect gut microbiota, they have limits. Mice have different gut microbes and body systems than humans, so the results may not fully apply. The diets used in the study are simpler than real human diets. Also, antibiotics were used to trigger infection, which does not match all human cases [68]. In addition to these, the growth of *Akkermansia muciniphila* and butyrate-producing taxa, like *Faecalibacterium*, was promoted through increased intake of complex carbohydrates. The fermented products produced by these microorganisms serve as mitochondrial fuel and epigenetic regulators. Since butyrate can inhibit histone deacetylases, activate gut-expressed G protein-coupled receptors, and help preserve epithelial integrity, aging pathways and a healthy lifespan are promoted. However, while animal and cellular studies suggest that fiber-derived SCFAs influence pathways related to longevity, mechanistic evidence directly linking fiber intake to human lifespan is still limited [69].

Garlic, bananas, and chicory root are rich in microbiota-accessible carbohydrates (MACs). The systemic relevance of MACs, including inulin, oligofructose, raffinose, and cellulose, was demonstrated by a study that indicated that MACs promoted saccharolytic fermentation and enriched *Lactobacillus* and *Faecalibacterium prausnitzii*. Further, it emphasized that improvement in gut barrier integrity, reduction in inflammation, and modulation of insulin sensitivity and lipid profiles were observed by the action of MACs through promoting the production of SCFAs. Additionally, an increment in *Bifidobacterium* species and suppression of *Clostridium* clusters were observed due to inulin supplementation. Further, the study demonstrated that an irreversible reduction in microbial diversity and mucus layer thickness can occur due to long-term fiber deprivation, even though fiber reintroduction is performed later. This fact highlights that consistent dietary intake is important [70].

### 6.2. Fats

Fats are essential macronutrients that supply energy, support cellular membrane structure, and regulate both metabolic and inflammatory pathways. Dietary fats are broadly categorized according to their chemical structure. Mainly, they are categorized as saturated fats and unsaturated fats. The unsaturated fats are then further categorized as monounsaturated (MUFAs) and polyunsaturated fatty acids (PUFAs). The physiological impact of these fats and their effect on gut microbiota is determined by these structural changes [71].

Western diets are typically characterized by high intake of saturated fats. Unfavorable microbial and metabolic outcomes have been consistently linked to this high intake of saturated fats. The promotion of gut dysbiosis, linked to reduced microbial diversity and a decline in beneficial bacteria, like *Bifidobacterium*, *Faecalibacterium*, and *Lactobacillus*, is the main disadvantage of these saturated fat-rich diets. On the other hand, the growth of pro-inflammatory taxa such as *Clostridium*, *Prevotella*, and *Enterococcus* is promoted by these diets while impairing gut barrier function and increasing systemic inflammation [72,73]. In a study related to maternal nutrition, the subjects were fed a high-fat lard-based diet during pregnancy and lactation. The results showed persistent alterations in the offspring’s gut microbiota, including increased *Firmicutes* and *Lachnospiraceae*, larger adipocytes, VAT fibrosis, and elevated inflammatory markers. However, these negative effects were observed to be reversed by supplementing a diet rich in omega-3-rich flaxseed oil. There, the production of beneficial microbial metabolites, like Hippurate, increased, thereby enriching the *Clostridium*, *Oscillospira*, and *Rikenellaceae* species [74].

When considering unsaturated fatty acids, especially when fish and plant oils rich in omega-3 polyunsaturated fatty acids (PUFAs) are consumed, they exert protective effects on both the gut and the brain, but not all studies agree on high-fat diet effects, and the context of overall diet and baseline microbiota matters. The growth of beneficial microbes, including *Bifidobacterium*, *Roseburia*, and *Faecalibacterium prausnitzii*, has been shown to be promoted by omega-3 fatty acids while enhancing mucosal barrier integrity and reducing gut-derived endotoxin levels, including lipopolysaccharides (LPSs) [72,73,75]. Other than that, improvement of metabolic regulation, cognitive function, and reduction in inflammation have also been observed through modulation of the gut–brain axis while reducing the risk of age-related cognitive decline.

In a study, after six weeks of consuming a modified Mediterranean ketogenic diet (MMKD), older adults with mild cognitive impairment exhibited significant changes in gut microbiota composition, including increased abundance of *Enterobacteriaceae*, *Akkermansia*, *Slackia*, and *Christensenellaceae*, and decreased levels of *Bifidobacterium* and *Lachnobacterium* [76].

Long-term consumption of high-fat diets, especially those rich in saturated fats, leads to gut dysbiosis characterized by reduced levels of beneficial bacteria, like *Bifidobacterium* and *Akkermansia*, and increased pro-inflammatory species, such as Firmicutes and Proteobacteria. These changes contribute to systemic inflammation and metabolic disorders. In contrast, short-term dietary interventions, even brief exposure to emulsifiers or high-fat meals, can already reduce microbial diversity and compromise gut barrier integrity. The type of fat also matters. Unsaturated fats promote microbial balance and anti-inflammatory effects, while saturated fats consistently disrupt microbial richness and elevate inflammation-related taxa. Chronic inflammation driven by these microbial shifts accelerates aging by promoting immunosenescence and tissue degeneration [13].

### 6.3. Proteins

Despite the source, animal and plant proteins play a key role in shaping gut microbiota-mediated outcomes associated with aging in different ways. Red meat and dairy, prominent sources of animal proteins, enhance the production of trimethylamine (TMA), which is then converted into trimethylamine-N-oxide (TMAO), a metabolite linked to cardiovascular disease and metabolic aging. But, while promoting the production of SCFAs, plant proteins support lipid regulation, microbial diversity, and potentially healthier aging trajectories [77,78].

Proteins and their derivatives, like peptides and hydrolysates, possess prebiotic potential. By skipping the full digestion in the upper gastrointestinal tract, these compounds reach the colon and promote the growth of beneficial microbes by serving as nitrogen-rich substrates. The viability, acid resistance, and enzymatic activity of *Lactobacillus* and *Bifidobacterium*-like species are enhanced by protein-derived oligopeptides from whey, casein, and soy. This is one of the examples that proves proteins’ ability to support a favorable microbial profile during aging [79].

Pulses are also a type of plant-based protein source that contain fermentable fibers and resistant starch. These pulse-based proteins promote the growth of longevity-associated taxa. Pulses are further considered as a sustainable source of protein associated with gut-centered strategies in aging due to their ability to increase SCFA production [80]. Proving the above fact, a study reports that red lentils, which are rich in protein, non-digestible carbohydrates, and polyphenols, have enhanced gut microbial diversity and SCFA levels in mice, specifically increasing *Roseburia*, *Prevotella*, and *Dorea*, which are linked to gut health and resilience [81].

Even though plant proteins are beneficial, not all plant proteins exert the same level of benefits for each individual. In line with this, a study demonstrated that glycated lentil proteins showed donor-specific shifts in microbial composition rather than significantly affecting the metabolite output [82].

However, animal proteins, mainly high-protein diets rich in sulfur-containing amino acids, have shown an increase in microbial production of hydrogen sulfide (H_2_S). H_2_S influences gut barrier disruption and metabolic stress associated with aging. Although short-term dietary changes in sulfur intake did not significantly alter sulfate-reducing bacteria, like *Desulfovibrio* and *Bilophila*, individual microbial stability appeared to mediate the response, suggesting that inherent microbiota composition may modulate the effects of dietary sulfur [83].

However, translating animal studies on protein intake and gut microbiota to human health presents key challenges. Differences in microbial composition, digestive physiology, and immune responses between species limit direct applicability. Animal models often use purified proteins and exaggerated doses, which do not reflect human dietary complexity. Additionally, microbial metabolites, like TMAO and ammonia, may behave differently in humans. Environmental, genetic, and lifestyle factors further complicate extrapolation, underscoring the need for well-controlled human trials to validate mechanistic insights.

### 6.4. Micronutrients and Bioactive Compounds (Vitamins, Polyphenols, Minerals)

#### 6.4.1. Vitamins

Vitamins use a highly specific set of interactions to shape the composition and functionality of gut microbiota. The synthesis and utilization of B vitamins (B1, B2, B3, B5, B6, B7, B9, and B12) are performed by specific microorganisms in the gut. These vitamins play a key role in energy metabolism and immune regulation, serving as essential coenzymes. Further, the presence of these vitamins promotes the growth of beneficial taxa like *Faecalibacterium*, *Bifidobacterium*, and *Roseburia* [84].

Other than vitamin B, the gut microbes produce menaquinones, a K2 form of vitamin K. This also plays a key role in regulating and modulating the gut microbiota. The growth of several taxa, such as *Faecalibacterium*, *Akkermansia*, and *Lactobacillus*, is promoted by these vitamins, and gut dysbiosis and compromised intestinal barrier function can be caused due to deficiencies or imbalances in K2, leading to dysfunctionalities in anti-inflammatory, antioxidant, and anti-aging pathways. However, despite severe deficiencies in certain vitamins, such as A, D, or B vitamins, which have been linked to dysbiosis and impaired intestinal barrier function in general, healthy populations, micronutrient effects appear more subtle [85].

Other than vitamins B and K, vitamins, such as C, E, and D, and carotenoids, like beta-carotene, lutein, and zeaxanthin, have also shown an effect on modulating gut microbiota composition. These compounds suppress the gut dysbiosis and related inflammation by promoting the growth of beneficial taxa, including *Bifidobacterium*, *Lactobacillus*, and *Faecalibacterium prausnitzii.* This microbial modulation contributes to retinal health and delays age-related macular degeneration through gut–retina axis regulation [70]. A study demonstrated similar effects by supplying the older subjects who are suffering from age-related macular degeneration with lutein, zeaxanthin, vitamins C and E, and zinc and suggested that those compounds restore gut microbial diversity and reduce inflammatory medium-chain fatty acids. Further, they increased the production of SCFAs that are beneficial to both gut integrity and eye health by increasing beneficial taxa, including *Faecalibacterium*, *Lachnospira*, and the *Eubacterium eligens* [86].

Another key nutrient that is vital for maintaining gut epithelial integrity is vitamin A. It promotes the production of immunoglobulin A (IgA) through gut-associated lymphoid tissue, thereby enhancing mucosal immunity [87].

However, intakes of water-soluble vitamins, such as vitamin B and C, have been associated with specific microbial shifts. They play a key role in maintaining metabolic and immune stability by correlating positively with *Bacteroides*. In contrast, intake of higher levels of fat-soluble vitamins A and D was negatively associated with *Lactobacillus.* This suggests that certain micronutrients may selectively suppress or promote probiotic species, depending on the aging gut environment [88].

#### 6.4.2. Minerals

Gut microbiota is influenced by minerals such as zinc, calcium, phosphate, and iron. These influences can change with age or health context. The intake of minerals affects microorganisms in a time-sensitive manner. As an example, a study that supplied zinc (6 ppm marginally deficient, 30 ppm adequate, and 300 ppm supplemented) for both young (2 months) mice and older (24 months) mice with zinc deficiency for 6 weeks demonstrated that in young mice, the gut microbiota was significantly altered, while in older mice, it was not that significant. Hence, age had shown a dominant influence on microbial composition, overriding zinc-related effects [89].

When fed with a high-fat diet, calcium supplementation also influences the gut microbiota by promoting the growth of beneficial genera, such as *Bifidobacterium* spp. and the *Bacteroides/Prevotella* ratio, while reducing *Clostridium coccoides* and *Clostridium leptum*. These shifts suggest that the negative shifts in the gut microbiome due to high-fat diets may partially be counteracted by the intake of calcium [90].

People with chronic kidney disease often require phosphate binders to manage high phosphate levels. Lanthanum carbonate is a phosphate binder, which reduces intestinal phosphate availability, leading to reduced abundance of phosphate-dependent species. Thereby, phosphate plays a role in shaping gut microbial structure. The growth of Actinobacteria was promoted through the treatment with lanthanum carbonate, while it suppressed the growth of *Parvimonas* and *Clostridium*. Phosphate can benefit the gut microbiome when supported by macronutrients. Fiber and complex carbohydrates enhance phosphate’s microbial utility by promoting SCFA-producing bacteria. Balanced protein and fat intake helps regulate phosphate availability, maintaining microbial diversity [91].

However, detrimental effects can occur on the gut microbiota through the excessive intake of iron. A study observed increased levels of *Defluviitaleaceae*, *Ruminococcaceae* UCG-014, and *Coprococcus* 1 in rats (n = 10 per group) who were fed with high doses of liquid iron (24 mg Fe by oral gavage daily for 30 days) and a drop in the count of *Lachnospiraceae FCS020* group and *Allobaculum*. Due to these alterations, oxidative stress, compromised barrier integrity, and heightened intestinal permeability resulted, indicating the potential risks of excess intake of iron on gut health. When paired with fiber, unsaturated fats, and plant proteins, iron supports gut health by promoting beneficial bacteria, reducing oxidative stress, and enhancing barrier integrity. These macronutrients modulate iron absorption and microbial balance, counteracting potential toxicity and fostering a resilient, anti-inflammatory gut environment that protects against iron-induced damage [92].

#### 6.4.3. Polyphenols

Polyphenols are different plant-derived compounds that contain antioxidant and anti-inflammatory properties. They can modulate the gut microbiota to promote healthy aging. Several studies have shown their ability to promote the growth of beneficial bacteria, such as *Bifidobacterium*, *Lactobacillus*, *Akkermansia muciniphila*, and *Faecalibacterium prausnitzii*, while suppressing potentially harmful taxa, like *Clostridium perfringens* and *Enterobacteriaceae*. The polyphenols support maintaining metabolic health and delaying age-related decline through enhancing intestinal barrier integrity, reducing oxidative stress and inflammation [2,93,94,95,96].

Higher polyphenol intake can maintain microbial resilience in aging populations through the promotion of the growth of beneficial taxa, such as *Bacteroides* and *Clostridium* cluster XIVa [88].

There are some polyphenols, such as curcumin, ellagic acid, EGCG, flavan-3-ols, and anthocyanins, which are not absorbed much in the small intestine. These compounds are transferred to the colon, where *Bifidobacterium longum*, *Enterococcus faecalis*, *Eggerthella lenta*, *Akkermansia muciniphila*, and *Clostridium coccoides*-like microbes convert them into bioactive compounds, such as urolithin A, phenyl-γ-valerolactones, and demethylcurcumin. These bioactive compounds are important to enhance antioxidant capacity, preserve cognitive function, modulate inflammation, and improve endothelial health [97,98,99]. Other than acting as a bioactive compound, culinary herbs and spices such as curcumin, luteolin, and quercetin can play a role as prebiotics while enhancing the growth of beneficial taxa [96].

However, across micronutrient and bioactive compound studies, there is considerable heterogeneity in design and methodology. Some studies are based on animal models, while others use human subjects, and there are large variations in nutrient type, dose, duration of intervention, and age or health status of participants. These differences make direct comparison challenging and can partly explain inconsistent findings across studies. Therefore, results should be interpreted with caution, recognizing that methodological diversity may limit the ability to draw uniform conclusions about micronutrient–microbiota interactions.

### 6.5. Probiotics and Prebiotics

#### 6.5.1. Probiotics

Probiotics are live microorganisms that live in human guts. When administered in adequate amounts, they can provide health benefits to the host. *Lactobacillus rhamnosus*, *L. acidophilus*, *L. casei*, *L. reuteri*, *Lactococcus lactis*, *Bifidobacterium bifidum*, *Bifidobacterium* spp., *Enterococcus*, *Akkermansia muciniphila*, and *Saccharomyces boulardii* are some common probiotic microorganisms. Through modulating the gut microbiota, these organisms maintain intestinal homeostasis while supporting immune regulation and reinforcing the gut barrier by reducing opportunistic pathogens and sustaining SCFA-producing taxa [100,101,102].

Competitive exclusion of pathogens, antimicrobial compound production (e.g., bacteriocins), and secretion of metabolites, like lactic acid and butyrate, are the mechanisms that probiotic microorganisms use to shape gut microbiota. For instance, *L. rhamnosus* GG produces specific bacteriocins against enteric pathogens, while *Bifidobacteria* enhance acetate output that protects against enteropathogens. A reduction in intestinal pH, inhibition of pathogenic biofilm formation, and regulation of inflammatory mechanisms are promoted by these microbes [100,101,102].

Upregulation of tight junction proteins, increased mucin and defensin production, and stimulation of secretory IgA-like mechanisms are used by probiotics to enhance barrier effects. Through these changes, epithelial integrity is fortified, and the intestinal permeability is reduced. As shown in the studies, *Akkermansia muciniphila* strengthened mucus thickness and *Saccharomyces boulardii* increased IgA responses [100,102].

Microbial diversity and composition can be modulated by probiotics. *Lactobacillus acidophilus*, *L. casei*, *L. rhamnosus*, *and Bifidobacterium bifidum* can modulate in separate pathways. *L. acidophilus* enhances gut health by producing bacteriocins and lactic acid, which inhibit pathogens. It supports epithelial integrity and promotes colonization of beneficial microbes in the small intestine. *L. casei* modulates immune responses and boosts microbial diversity. It helps suppress harmful bacteria and supports the growth of commensals, especially under stress or antibiotic-induced dysbiosis. *L. rhamnosus* strengthens mucosal barriers and regulates cytokine production. It promotes the resilience of gut flora, reduces inflammation, and supports recovery from microbial imbalance caused by diet or infection. *Bifidobacterium bifidum* ferments complex carbohydrates into short-chain fatty acids, enhancing microbial richness. It inhibits pathogens, supports immune modulation, and maintains gut homeostasis, especially in aging or compromised hosts. Further, the metabolic parameters had improved while the circulation of lipopolysaccharides had been suppressed [103]. Additionally, probiotics have promoted immune resilience and reduced systemic inflammation in infants and animal models by modulating microbial evenness [102,104].

#### 6.5.2. Prebiotics

The growth and activity of beneficial gut microbes can be enhanced by prebiotics, which are selectively fermented substrates for the improvement of gut health. Inulin-type fructans (ITFs), oligofructose, galactooligosaccharides (GOSs), resistant starch, and aloe polysaccharides (APs) are broadly studied compounds that have proven their ability to enhance the gut microbial composition by promoting the growth of *Bifidobacterium*, *Faecalibacterium prausnitzii*, and clostridial cluster XIVa while decreasing the levels of Firmicutes and *Enterobacteriaceae*. These compounds engage in several mechanisms to enhance short-chain fatty acid (SCFA) production, suppress inflammation, promote gut homeostasis along with gut integrity, increase immunity, and maintain metabolic function [48,105,106,107,108,109,110].

When considering ITFs, they have demonstrated their ability to select specific gut microorganisms, including *Bifidobacterium* spp., to support the reversal of gut dysbiosis and systemic lipopolysaccharides in mouse models that are suffering from diabetes and obesity [48]. In line with this, another study has fed obese adults with inulin (16 g/day) for 3 months and found that the supplementation has promoted the growth of *Bifidobacterium* and *Haemophilus* microorganisms in individuals with a high baseline abundance of *Coprococcus*. However, the article notes an increase in *Haemophilus* after inulin supplementation, but this genus includes both helpful and harmful species. For example, *H. parainfluenzae* may be harmless, while *H. influenzae* is pathogenic. Since the study does not specify which species were observed, it is best not to assume all *Haemophilus* are beneficial [106].

In addition to ITFs, supplementation of GOS has also demonstrated an increase in Bifidobacterium abundance in both in vitro and in vivo models, indicating it as a promising method for maintaining healthy aging. This alteration in microbiota has led to elevated butyrate production while indicating the improved fermentative balance, reduced gut toxicity and metabolic resilience [110]. Upregulation of genes involved in mucus production, goblet cell function, and mucin glycosylation is considered a main factor in maintaining epithelial barrier integrity. Oligofructose, by promoting the growth of beneficial genera, such as *Akkermansia*, *Odoribacter*, and *Muribaculaceae*, maintains gut barrier integrity by regulating the above-mentioned factors. Further, it supports microbial fermentation and thereby acts as a potential prebiotic [109].

Supplementation of aloe polysaccharide (AP) has been shown to improve epithelial integrity and immune modulation in a mouse model study. In its mechanism, an enhancement of the abundance of SCFA-producing genera, such as *Bacteroides* and *Parabacteroides*, and a reduction in the count of *Firmicutes* and *Clostridium*, were observed. During the microbial shift, the production of acetate, propionate, and butyrate was promoted [108].

## 7. Gut Microbiota and Aging: Mechanistic Insights

**Figure 4:**
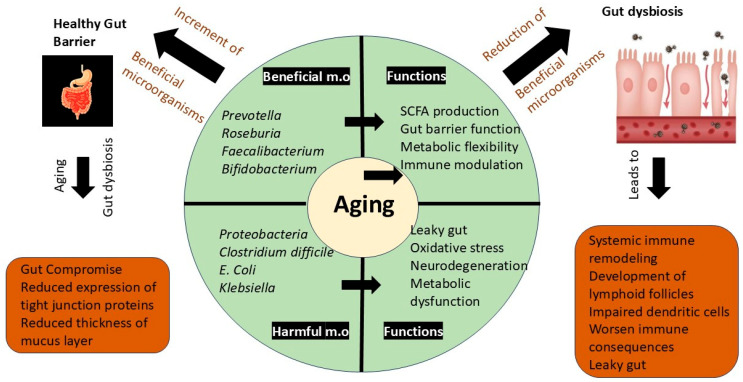
Effects of beneficial and harmful bacteria on gut health. SCFA-Short chain fatty acids, m.o-microorganisms.

Gut dysbiosis and the weakening of host–microbiota mutualism can be caused due to aging-related physiological changes, where the abundance of beneficial microorganisms, like *Clostridium* cluster XIVa, and the production of SCFAs that are needed for maintenance of immune, cognitive health, and metabolic activities, are reduced. Biological, clinical, and psychological changes may occur during aging that affect gut health accordingly [111] (Figure 4).

### 7.1. Mechanisms of Gut Microbiota–Aging Interaction

Exposure of gut microbes from early childhood to elderly life shapes the development of the immune system, mucosal tolerance, and the regulatory function of lymphocytes. This lays a foundation for long-term beneficial health outcomes [112]. Even though the early stages of life have better compositional changes in the microbiome, when a person ages, the relationship becomes dysregulated, leading to immune dysfunction. With aging, beneficial anaerobes, like *Faecalibacterium prausnitzii* and *Bifidobacteria*, tend to decline while facultative anaerobes, such as Enterobacteriaceae, increase [113,114].

The gut dysbiosis related to aging may cause different losses, including anti-inflammatory strains, such as *Faecalibacterium prausnitzii*, *Eubacterium hallii*, and *Roseburia*, and diminished regulatory T cell output. Due to these compositional changes, systemic immune remodeling is accelerated through senescence-associated secretory phenotypes [115]. Further, these disruptions cause the development of lymphoid follicles, an intestinal immune structure, thereby interfering with the feedback loop between microbiota and immune maturation, including impaired dendritic and regulatory T cell development [116]. The loss of *Bacteroidetes*, *Lactobacillus*, and *Bifidobacteria* due to aging can further worsen the immune consequences associated with aging.

The spatial gradients of the gastrointestinal tract, including pH, oxygen, and mucus levels, tend to change when the hist ages. The niche specialization of anaerobic bacteria, such as *Faecalibacterium* and *Lachnospiraceae*, gets affected by these changes, leading to weakened microbial–host signaling required for nutrient metabolism, immune coordination, and endocrine responses, especially in the small intestine [117].

Leaky gut is one key manifestation of this disruption. When the gut becomes leaky, the intestinal barrier becomes compromised, allowing the passage of pathogens, toxins, and undigested food into circulation. Upregulation of Zonulin, a tight junction regulator, happens due to the microbial imbalance occurring with aging, thereby weakening epithelial barriers and activating immune responses, such as T helper 17 cell differentiation. These changes may fuel different illnesses with aging, mainly chronic inflammation, type 1 diabetes, and celiac disease [118,119].

Frailty is a condition marked by reduced physiological reserve and increased vulnerability to stress. This has proven to be associated with compositional changes of the gut microbiome. A study demonstrated that lower levels of Lactobacilli, *Bacteroides*/*Prevotella*, and *Faecalibacterium prausnitzii* have been identified among older adults with different levels of frailty, while they showed elevated counts in Enterobacteriaceae [120]. Hence, microbial composition may both reflect and influence frailty status in aging populations [121].

### 7.2. Gut Barrier Function and Permeability

The gut barrier consists of several layers that maintain intestinal integrity and regulate host–microbe interactions. The layers, namely, are the mucus layer, epithelial cell layer, and immune components in the lamina propria. These three layers act together to provide physical protection and immunological communication in the gut. The goblet cells secrete MUC2 mucin to compose the mucus layer. The mucus layer comprises two sub-layers in the colon, which are stratified, where the inner layer is a dense layer free of microbes and the outer layer is a loose layer colonized by commensals. Under this layer, another layer called the epithelial monolayer can be seen. Enterocytes, goblet cells, Paneth cells (in the small intestine), enteroendocrine cells, tuft cells, and microfold (M) cells are the components of this epithelial monolayer. Absorption of nutrients and the secretion of antimicrobial peptides and sample luminal antigens are the main roles of this layer. Tight junctions, adherens junctions, and desmosomes together seal the intercellular junctions while regulating the tissue permeability. Lamina propria, which contains immune cells, such as dendritic cells, macrophages, and lymphocytes, as well as organized lymphoid structures, like Peyer’s patches, supports this physical structure of the epithelial monolayer. Therefore, the gut barrier is a main dynamic interface between host immunity and microbial populations, which coordinate immune responses to promote tolerance or defense [122].

This complex barrier system tends to compromise with aging. In line with this, a mouse model study has demonstrated a six-fold reduction in colonic mucus thickness, leading to an increased microbial contact with the epithelial surface. The expression of tight junction proteins such as occludin, ZO-1, and TJP2 has also been shown to be reduced [114,123].

Another factor that impairs the gut barrier function is microbial dysbiosis. *Faecalibacterium prausnitzii* is considered a beneficial taxon that produces butyrate, which is crucial for maintaining tight junction integrity and epithelial energy supply. Dysbiosis may lead to a reduction in the abundance of these taxa [124]. *Lactobacillus royi* is also such a beneficial taxon that contributes to reducing microbial support for epithelial structure and function [113].

Restoring microbial balance by therapeutic interventions is considered a remedy to rejuvenate barrier integrity. A study that was performed using aged mice identified an upregulation of TJP2 at both mRNA and protein levels and an increment in goblet cell counts and enhanced mucus thickness through the intervention of *Gastrodia elata* and its active compound parishin, suggesting a reinforcement of both the chemical and physical aspects of the barrier [125].

However, epithelial integrity and alleviating age-related pathology can be improved by transplanting microbiota from young donors, confirming a causal role of gut microbial balance in preserving the gut barrier during aging [126].

### 7.3. Production of Metabolites and Their Effects on Aging

Fibers, bile acids, and amino acids that are transferred from the intestine to the gut are metabolized into bioactive compounds that modulate host physiology by gut microbiota. The most important among them are short-chain fatty acids (SCFAs) and bile acid derivatives. These compounds influence epithelial renewal and immune responses [111]. The disruption of microbial metabolism with aging happens particularly through the decline of SCFA-producing bacteria, such as *Clostridium* clusters IV and XIV and *Bifidobacterium* [114]. Other than that, with aging, the occurrence of saccharolytic metabolism reduces while it increases the proteolytic metabolism, thereby reducing SCFA levels while increasing less beneficial metabolites. As an example, reduced production of indole, a tryptophan-derived compound essential for epithelial renewal, is linked with decreased health span and elevated mortality risk.

Butyrate is an SCFA that utilizes different epigenetic mechanisms to influence host immunity. Butyrate has the ability to enhance the regulatory T cell differentiation and promote anti-inflammatory cytokines, such as IL-10, through the inhibition of histone deacetylases (HDACs). Through this action, epithelial regeneration improves, and chronic inflammation tends to decline, highlighting the immune-modulatory role of SCFAs in aging [126]. Further, anti-aging dietary interventions, such as calorie restriction and intermittent fasting, have been proven to enrich SCFA-producing bacteria, like *Lactobacillus*, *Bifidobacterium*, and *Roseburia*, which contribute to the improvement of beneficial metabolic output and may delay age-associated dysfunction [125].

Bile acid is also a microbial-derived metabolite. Primary bile acids are synthesized from cholesterol in the liver. These bile acids serve as signaling molecules once converted into secondary bile acids by gut microbes. In addition to that, they aid fat digestion. These secondary bile acids influence glucose metabolism, energy expenditure, gut barrier function, and inflammation by activating host receptors, like the farnesoid X receptor (FXR) and Takeda G protein-coupled receptor 5 (TGR5). Gut dysbiosis significantly shifts bile acid pools and signaling capacity, linking this axis to metabolic disorders, such as type 2 diabetes and liver disease [117].

## 8. Diet and Aging

### 8.1. Diet and Biology of Aging

Diet plays an important role in modulating the biological processes that drive aging, influencing longevity and disease risk through multiple molecular pathways. The Mediterranean diet has emerged as a model that promotes healthy aging by influencing both biological and clinical dimensions of the aging process. It has been shown to support longevity through epigenetic mechanisms such as DNA methylation, histone modification, and microRNA regulation. While being rich in bioactive compounds, such as polyphenols from olive oil and sulfur-containing molecules found in cruciferous vegetables, this dietary pattern helps regulate the cell cycle, mitigate oxidative damage, and temper key processes, like inflammation and carcinogenesis. The Mediterranean diet promotes longevity by reducing chronic disease risk. In the Seven Countries Study of 12,000 men (40–59 years), coronary heart disease incidence was much lower in Greece (32/10,000 person-years) and Yugoslavia (53/10,000) than in the U.S. (177/10,000) and Finland (198/10,000). The *PREDIMED* (short for Prevención con Dieta Mediterránea) trial (7018 participants) further showed that high adherence reduced stroke risk in genetically predisposed individuals to levels comparable with low-risk groups. A smaller interventional study (12 healthy subjects, 12 metabolic syndrome patients) confirmed that polyphenol-rich olive oil improved insulin sensitivity and gene regulation. Collectively, these findings link the Mediterranean diet to longer life through cardiovascular protection and molecular benefits [127]. The modified Mediterranean diet supports longevity and healthy aging by prioritizing plant-based foods, unsaturated fats, and modest alcohol intake. A multi-center prospective cohort study across nine European countries followed 74,607 adults aged ≥60 years who were free from coronary heart disease, stroke, or cancer at enrolment. Adherence to a modified Mediterranean diet was scored on a 10-point scale. Each two-point increase in the score was linked to an 8% reduction in overall mortality (95% CI: 3–12%), with consistent results across countries and stronger effects in Greece and Spain. After dietary calibration, the reduction remained 7% (95% CI: 1–12%), confirming that the Mediterranean diet promotes increased survival and longevity in older Europeans [128].

Oxidative stress plays a central role in vascular aging, largely by impairing endothelial function. As we age, the vascular lining becomes a key site of reactive oxygen species (ROS) production from sources like NAD(P)H oxidase and mitochondria, which degrades nitric oxide (NO) and forms harmful by-products, such as peroxynitrite. This redox imbalance contributes to vascular stiffness and dysfunction. Diets high in saturated fats worsen the issue by increasing oxidative and inflammatory stress, while those rich in polyphenols and unsaturated fats may help preserve NO availability and support healthy endothelial aging [129].

The Mediterranean diet may contribute to slow biological aging by positively influencing the DNA repair mechanisms and reducing the DNA damage due to oxidative stress. The Mediterranean diet’s rich content of bioactive compounds (melatonin, phytosterols, carotenoids, polyphenols, resveratrol, hydroxytyrosol, and glucosinolates) works together to preserve telomere length and enhance genomic stability. Correspondingly, individuals following this diet often exhibit lower levels of oxidative stress markers, like 8-OHdG, underscoring its potential protective role in the aging process. In a randomized crossover trial involving 20 hypercholesterolemic individuals, daily consumption of a Mediterranean-type meal rich in olive oil, fruits, and vegetables for four weeks significantly reduced plasma oxidized LDL levels by 25% compared to a Western-type diet, highlighting improved antioxidant defense. Similarly, an interventional study with 30 patients with metabolic syndrome reported that adherence to a Mediterranean diet supplemented with extra virgin olive oil for 12 weeks lowered malondialdehyde (MDA) concentrations by 22%, a key marker of lipid peroxidation. These findings suggest that the diet’s antioxidant-rich profile directly mitigates oxidative damage, contributing to healthier aging [130].

The CALERIE (Comprehensive Assessment of Long-term Effects of Reducing Intake of Energy) trial was the first randomized study to examine how cutting calories affects aging in healthy, non-obese adults. By reducing intake by 25% over two years, participants showed slower biological aging, marked by lower DNA methylation age and oxidative stress. Echoing earlier animal studies, the results suggest that restriction of high-calorie intake can improve mitochondrial function, curb inflammation, and delay aging [131].

A review underscores that plant-forward eating patterns like the Mediterranean, DASH (Dietary Approaches to Stop Hypertension), and MIND (Mediterranean-DASH Intervention for Neurodegenerative Delay) diets are linked to better physical and cognitive health. High adherence to these diets has been associated with slower memory loss, fewer mood issues, sharper senses, improved fitness, and reduced frailty. These benefits are largely credited to their anti-inflammatory, antioxidant-rich nature and strong overall nutritional balance [132].

### 8.2. Diet and Physical/Cognitive Function of Aging

Nutrition plays a key role in physical and cognitive health during aging, thereby preserving functional abilities. In particular, the Mediterranean diet itself is linked to reduced rates of neurodegenerative diseases, cardiovascular issues, and general frailty by enhancing the physical and cognitive function of people. As an example, the synergetic action of foods, such as tomatoes and olive oil, where enhanced bioavailability of lycopene can be observed when tomatoes are eaten along with olive oil, helps maintain metabolic and neurological health in aging individuals [127].

Long-term adherence to a nutrient-rich Mediterranean-style diet may help preserve functional capacity in older age. A dietary pattern rich in vegetables, fruits, and whole grains alongside moderate consumption of fish and dairy appears to support energy balance, nutritional sufficiency, and sustained mobility. This aligns with observed links between greater adherence to this diet and a lower risk of early mortality [128]. Traditional Mediterranean dietary patterns characterized by olive oil, vegetables, fish, and moderate wine intake have been linked to a lower risk of age-related neurodegenerative diseases, such as Alzheimer’s and Parkinson’s. These protective effects are largely attributed to neuroactive compounds, like omega-3 fatty acids, which may help slow cognitive decline and preserve memory and executive function in later life [64].

Evidence from the NU-AGE project suggests that adherence to a Mediterranean diet may support cognitive function in older adults, although effects can vary between individuals. In this multi-center, interventional study, 612 non-frail or pre-frail participants aged 65–79 from five European countries (UK, France, the Netherlands, Italy, and Poland) were assigned to a 12-month MD tailored to elderly subjects. Gut microbiota profiling before and after the intervention revealed that diet adherence increased the abundance of specific bacterial taxa, which were positively associated with markers of lower frailty and improved cognitive performance and negatively associated with inflammatory markers, such as C-reactive protein and interleukin-17. While the associations were observed independently of age or body mass index, individual responses may differ due to baseline microbiota composition and other host factors. Collectively, the findings suggest that dietary modulation of the gut microbiome via the Mediterranean diet has the potential to support cognitive health in aging, but benefits may not be uniform across all individuals [32].

#### Effect of DASH and MIND Diet

The DASH diet stands for dietary approaches to stop hypertension. It is designed to lower blood pressure and improve heart health. It emphasizes fruits, vegetables, whole grains, low-fat dairy, lean meats, nuts, and seeds. DASH diets limit sodium, red meat, sweets, and saturated fats. They are rich in potassium, magnesium, calcium, and fiber. On the other hand, the MIND diet stands for Mediterranean–DASH intervention for neurodegenerative delay. This combines elements of the Mediterranean and DASH diets, tailored to protect brain health. It emphasizes green leafy vegetables, berries, nuts, whole grains, fish, olive oil, and poultry, and it limits red meat, butter, cheese, pastries, sweets, and fried foods. Both of these diets are different from the Mediterranean diet (Table 3).

The Mediterranean, DASH, and MIND diets have all been shown in numerous studies to support cognitive development and healthy brain aging, with consistent evidence linking their nutrient-rich patterns to improved memory, slower cognitive decline, and reduced risk of dementia (Table 4).

The DASH diet supports healthy aging, primarily through cardiovascular protection. By lowering blood pressure and LDL cholesterol, it reduces stroke risk by up to 40% and slows atherosclerosis progression, while higher potassium and fiber intake improve vascular function and decrease inflammation, enhancing cardiovascular resilience in older adults [132]. These cardiometabolic benefits extend to brain health, with long-term adherence linked to better cognitive outcomes, including improved memory, reduced dementia risk, and higher MMSE scores by 1.3–2.1 points. Blood pressure reductions of up to 7.2 mmHg, declines in inflammatory markers by 20–35%, and gains in verbal memory and cerebral perfusion further highlight its neuroprotective role [134]. Evidence also indicates that DASH slows cognitive decline, particularly in processing speed and global cognition, though improvements in working memory are less consistent, especially in diverse aging populations [135]. While its anti-inflammatory properties may help delay Alzheimer’s disease by modulating systemic and neuroinflammation, these effects are strongest with long-term adherence or when combined with physical activity, emphasizing the importance of lifestyle integration for brain and vascular health during aging [136].

The MIND diet emphasizes neuroprotective foods that support brain aging, with consistent evidence linking higher adherence to better cognition. Meta-analyses show that each three-point increase in adherence raises cognitive scores by 0.110, an effect equivalent to being one year younger. These benefits are attributed to higher intake of nuts, fish, tea, berries, and leafy greens, alongside reduced fried food consumption, which collectively enhance memory and slow decline [143]. In middle-aged adults, the diet’s effects were evident in faster processing speed, reflected by reduced P3 latency (a key marker in cognitive neuroscience and psychology, especially in studies of attention and decision-making) during demanding attention tasks, suggesting enhanced neural efficiency not observed with other dietary patterns [137]. In older adults at risk for dementia, the MIND diet was associated with modest improvements over three years; however, outcomes were not significantly greater than those observed in a control group following caloric restriction, with both groups showing similar cognitive and brain volume changes [138]. These findings suggest that while adherence to the MIND diet can support cognitive health and efficiency, its distinct neuroprotective advantages may be context-dependent and require longer-term evaluation in aging populations.

The MIND diet, designed to mainly promote brain health, combines key elements of the Mediterranean and DASH diets, placing special emphasis on leafy greens, berries, whole grains, nuts, and fish while limiting red meat, butter, and sugary treats. In a study, individuals who closely followed this diet experienced significantly slower cognitive decline over nearly five years. Remarkably, those with the highest adherence performed cognitively as if they were 7.5 years younger. This association is held even after accounting for lifestyle, health, and genetic risk, underscoring the diet’s potential to protect against age-related cognitive decline [26].

A well-balanced vegetarian diet may play a key role in healthy aging through several mechanisms. This helpful diet pattern can lead to a lower body mass index (BMI), reduce total and LDL cholesterol, improve blood pressure, and enhance insulin sensitivity, factors that synergistically enhance cardiovascular and metabolic stability in older adults. Increasing the intake of antioxidants, fiber, phytoestrogens, and omega-3 fatty acids from plant-based foods can protect against oxidative stress and age-related tissue damage, which are considered the hallmarks of biological aging. The reduction in markers of inflammation due to a vegetarian diet may further contribute to preserving vascular and cellular health. The vegetarian diet may help maintain energy balance, reduce frailty, and support mobility when well-planned. However, to gain these aging-related benefits, individuals must have an adequate intake of protein, vitamin B12, iron, zinc, and omega-3s, nutrients that are less abundant in plant-based foods [27].

## 9. The Interconnected Triangle: Diet, Gut Microbiota, and Aging

**Figure 5:**
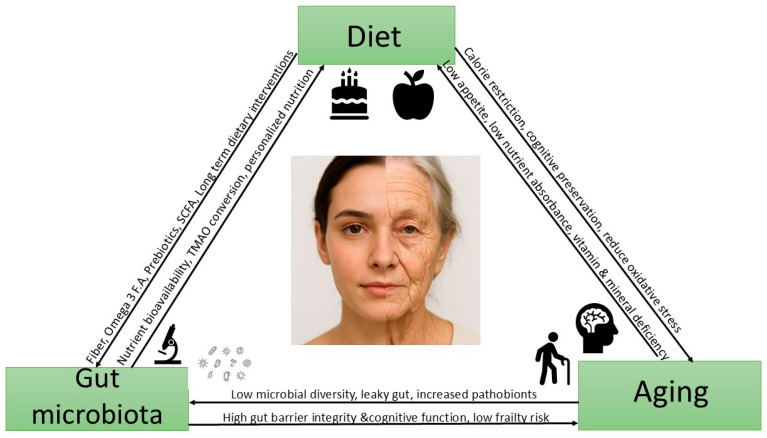
The interconnected triangle among diet, gut microbiota, and aging (“The human face element in this figure was generated with the assistance of ChatGPT (OpenAI, San Francisco, CA, USA Available at: https://chat.openai.com/, accessed on 7 July 2025)”.

Aging is associated with diversified changes in both dietary patterns and gut microbiota composition, and these three elements, diet, gut microbiota, and aging, are tightly interconnected, each influencing and being influenced by the others in a dynamic triangular relationship (Figure 5).

### 9.1. Interconnection of Gut Microbiota and Aging

The gut microbiota can positively affect aging by regulating immune responses, maintaining gut barrier integrity, and producing anti-inflammatory metabolites, like short-chain fatty acids (SCFAs). Among these SCFAs, butyrate is significantly important for diminishing systemic inflammation, promoting colon health, and modulating brain function. Dysbiosis or change in the microbial composition in the gut in older adults tends to increase gut permeability and chronic inflammation, which also contribute to neurodegenerative diseases, like Alzheimer’s and Parkinson’s. Furthermore, changed microbiota can impair brain-derived neurotrophic factor (BDNF) expression and synaptic plasticity, negatively impacting memory and cognition [144].

Host physiology is further affected by gut microbiota towards enhancing aging. The key functions related to this are biosynthesis of vitamins (e.g., thiamine, niacin), metabolism of carbohydrate and fiber, and development of the immune system. During childhood, species like *Bifidobacterium longum* predominate, and they can support nervous and immune development via vitamin production. With aging, microbial enzymes that are involved in metabolizing fiber become more dominant, mainly due to dietary changes, and they possibly help regulate inflammation and metabolic health [145].

An increase in Gram-negative bacteria is observed with aging, which decreases microbial diversity. The release of lipopolysaccharides by these bacteria tends to enhance inflammation. Reduced fiber intake, antibiotic use, and immune-senescence are the factors that contribute to this shift. As a result, the production of beneficial SCFAs is reduced and immune and metabolic functions get impaired, which accelerates aging in return [144].

Furthermore, some core gut species, such as *B. longum*, tend to decline, while microbes in taxa like *Faecalibacterium*, *Roseburia*, and *Ruminococcus* may rise with advancing age. Additionally, *Akkermansia* and *Clostridium* cluster IV, like some beneficial microbiota, also show a drop in count, thereby notably weakening both immune and metabolic functions. Therefore, both compositional and functional changes are considered to be associated with aging, which changes the gut microbiota ecosystem [145].

### 9.2. Interconnection of Diet and Aging

A beneficial gut microbial composition is enhanced by high levels of fiber. Gut microbes can ferment these dietary components into short-chain fatty acids (SCFAs), like butyrate, which are essential for the regulation of immune responses, maintenance of gut barrier integrity, and reduction in inflammation, which are considered the key factors affecting healthy aging [126]. On the other hand, Western diets that are rich in saturated fats, red meat, and low in fiber lead to dysbiosis in gut microbial composition. As a result of this imbalance, the production of harmful metabolites, like trimethylamine-N-oxide (TMAO), is enhanced, thereby increasing gut permeability (“leaky gut”), and chronic inflammation is a main factor attributed to accelerated aging [146].

However, age-related oxidative stress and inflammation, the drivers of cellular aging, can be reduced using a vegetarian diet that is rich in antioxidants, mainly vitamin C, carotenoids, and polyphenols. Reduced DNA damage, lipid peroxidation, and higher plasma antioxidant concentrations have been observed in elderly people who have followed a vegetarian diet, suggesting that diet can mitigate oxidative damage typically associated with aging. Further, a healthier gut function can be promoted by the high antioxidant intake from plant-based foods while preserving physical and cognitive function in older ages [147].

While diet can influence aging, on the other hand, aging also influences diet through biological and behavioral changes. Insulin signaling, mitochondrial efficiency, and nutrient sensing (like mTOR and AMPK-like mechanisms) in the human body tend to shift with age, affecting the way food behaves inside the body. Restriction of high calories is a promising method for elderly people to endure these metabolic changes. Furthermore, appetite, taste, and physical activity can also be affected by aging, while altering personal food choices and nutrient intake suggest that personalized diets are important for promoting health and longevity in later life [131].

Refined grains, sugars, saturated fats, and low fiber are characteristic dietary components of a Western diet. These components disrupt microbial diversity while leading to gut dysbiosis and thereby enhance the pro-inflammatory species. An increase in intestinal permeability (“leaky gut”) is another function of these dietary components, where they allow microbial products, like lipopolysaccharides (LPSs), to enter circulation and trigger systemic inflammation. On the other hand, the growth of beneficial microbes that produce anti-inflammatory short-chain fatty acids (SCFAs), such as butyrate, is promoted by vegetarian diets that are rich in unprocessed plant foods, fiber, and polyphenols (like ancestral or Paleolithic diets) [4,148].

A study demonstrates that the regulation of host energy harvest and nutrient availability is mainly influenced by gut microbiota. The breakdown of complex carbohydrates and the increase in absorption of short-chain fatty acids are performed by certain microbial communities, thereby contributing to energy extraction from otherwise indigestible fibers. Other than that, satiety hormones and lipid metabolism are also subtly shaped. Therefore, rather than just responding to diet, the gut microbes actively shape how the bodies process and respond to what is consumed [4].

Supporting the previous fact, another study showed that the way nutrients are metabolized and the way our body responds to dietary components are typically influenced by the gut microbiota. Proving this fact, it has been identified that the digestion of complex polysaccharides and the synthesis of vitamins (like B and K vitamins) are enhanced by beneficial species. However, dysbiotic microbiota promote chronic low-grade inflammation, insulin resistance, and oxidative stress by impairing nutrient metabolism, which suggests that even a moderate diet can be more harmful to the host [145].

## 10. Research Gaps and Future Research Directions

Despite substantial progress, several gaps remain in our understanding of the complex relationship between diet, gut microbiota, and aging. A major limitation is the scarcity of longitudinal, age-stratified human intervention studies. Most available evidence is derived from short-term trials or animal models, which may not capture the full trajectory of microbiota shifts and their impact on aging over time. Furthermore, elderly individuals, particularly those in institutional care or with frailty, are underrepresented in current research, limiting the generalizability of findings to this vulnerable population.

Another key challenge is the lack of standardization in microbiome sampling, sequencing, and data interpretation, which complicates cross-study comparisons and meta-analyses. Establishing consensus on core analytical pipelines and reference databases will be essential for generating reproducible, scalable insights. Moreover, many studies overlook inter-individual variability in microbiota responsiveness to diet, likely driven by host genetics, lifestyle, and baseline microbiota composition, underscoring the need for personalized approaches.

The integration of multi-omics technologies, including metabolomics, transcriptomics, and proteomics, offers a promising avenue to uncover mechanistic links between microbial metabolism and host aging phenotypes. However, such approaches remain underutilized in aging-focused nutrition research. Future studies should also explore the gut–organ axes (e.g., gut–brain, gut–muscle, gut–retina) in more depth to understand how microbial signals affect systemic aging processes.

Finally, there is limited insight into how early-life diet and microbiota may influence aging trajectories decades later, or whether microbiota-targeted interventions in youth or midlife could offer preventive benefits. Addressing these knowledge gaps through interdisciplinary, longitudinal, and population-diverse studies will be crucial for translating microbiome science into effective dietary strategies for healthy aging.

## 11. Conclusions

The interplay between diet, gut microbiota, and aging represents a dynamic and modifiable system with profound implications for human health. Aging is accompanied by notable shifts in gut microbial composition, including reduced diversity and the loss of beneficial taxa, which contribute to systemic inflammation, impaired immunity, and metabolic dysfunction. However, dietary patterns, especially those rich in fiber, polyphenols, and healthy fats, can reshape the microbiota, enhance production of beneficial metabolites, like short-chain fatty acids, and mitigate age-related decline. The Mediterranean, plant-based, and other nutrient-rich diets have shown promise in promoting microbial profiles associated with reduced frailty, preserved cognition, and improved metabolic health. Importantly, the gut microbiota functions not just as a target but also as a mediator, translating dietary inputs into molecular signals that influence host aging processes. Emerging evidence supports the potential of microbiota-targeted dietary interventions such as prebiotics, probiotics, and precision nutrition to promote healthy aging. Nonetheless, translating these findings into real-world solutions requires deeper mechanistic insights and broader clinical validation. By recognizing the gut microbiota as a key interface between nutrition and aging, future strategies may more effectively support longevity and functional health across the lifespan.

## Figures and Tables

**Table 1:** Dietary patterns, their metabolites, and the impact on gut microbiota and health.

Diet Pattern	Impact on Gut Microbiota	Production of Metabolites	Impact on Health	References
Mediterranean diet	Increased - *Prevotella* spp. - *Roseburia* spp. - *Lachnospira* spp.	- High amount of short-chain fatty acids (SCFAs)—propionate, acetate, butyrate - Low production of TMAO (trimethylamine oxide)	- Higher SCFAs—better gut barrier, lower chronic inflammation, reduced frailty - Greater *Prevotella* and diversity—support metabolic stability and delay functional decline	[22]
	- Increased Bifidobacteria to *E. coli* ratio - Decreased *Escherichia coli* counts - Increased relative abundance of *Bacteroides* species - Higher detection of *Candida albicans*	- Decreased valerate and other protein fermentation by-products	- Lower *E. coli* and higher *Bifidobacteria* support immune balance - Lower valerate suggests reduced protein fermentation and gut toxicity, beneficial for long-term gastrointestinal health - Overall microbiota profile aligns with better metabolic and anti-inflammatory status in aging	[23]
	- Increased abundance of *Faecalibacterium prausnitzii*, *Clostridium* cluster XIVa, and *Bacteroides* - Decreased Firmicutes/*Bacteroidetes* ratio with high adherence	- Increased synthesis of anti-inflammatory compounds via polyphenol metabolism	- Enhanced microbial richness supports gut barrier function and resilience to age-related dysbiosis	[24]
	- Enrichment of fiber-fermenting bacteria	- Lower production of harmful by-products, like lipopolysaccharides - Enhanced bile acid modulation	- Lower LPS (lipopolysaccharides) reduces systemic inflammation, potentially delaying age-associated diseases - Favorable gut ecosystem helps mitigate frailty	[18]
Western diet	Increased - *Ruminococcus* spp. - *Streptococcus* spp. - *Alistipes* spp.	- Trimethylamine N-oxide (TMAO) - Valerate and caproate - Succinate - Phenolic and indole derivatives (e.g., p-cresol, indoxyl sulfate) - Decreased short-chain fatty acids (SCFAs): acetate, propionate, butyrate	- Increased *Alistipes* and *Streptococcus*, which are linked to inflammaging and cognitive decline - Higher *Ruminococcus*—associated with increased oxidative stress in older adults - Succinate—associated with higher oxidative stress and metabolic imbalance	[22]
	- Reduced *Akkermansia* - Increased *Romboutsia* and *Parasutterella* - Overall shift toward pro-inflammatory microbial composition	- Increased endoplasmic reticulum (ER) stress markers - Elevated markers of oxidative stress in adipose and muscle tissue - Altered gut hormone profiles and insulin signaling pathways	- Lower *Akkermansia* and microbial richness may impair gut barrier - Increased ER and oxidative stress are linked to insulin resistance and muscle decline - Raise the risk of frailty and age-related disease	[25]
	- Increased presence of *Bacteroides fragilis* and *Clostridium difficile*	- Elevated reactive oxygen species (ROS)	- Increased oxidative stress, inflammaging, and immune senescence - Gut dysbiosis	[26]
	- Increased *Enterobacteriaceae* - Reduced *Lactobacillus* and SCFA-transporting bacteria - Shift toward *Firmicutes* and *Proteobacteria* - Altered balance of *Bacteroidetes* and *Firmicutes*	- Elevated sugar-derived metabolites that impair neuronal metabolism	- Elevated risk for neurodegeneration and age-related cognitive dysfunction - Altered microbiota memory impairments and mood decline in aging	[27]
	- Increased *Alistipes*, *Lachnospiraceae FCS020*, and *Coprococcus* - Rise in *Ruminococcaceae NK4A214* group and *Roseburia*	- Increased branched-chain amino acids (isoleucine, leucine, valine) - Raised microbial pathways tied to cholesterol and lipid metabolism	- Changes in microbial lipid-processing genes—contribute to early-onset dyslipidemia and liver stress	[28]
Plant-based diet	- Increased relative abundance of *Ruminococcaceae*, *Lachnospiraceae*, and *Akkermansiaceae* - Decreased levels of *Bacteroides fragilis* and other meat-associated taxa - No major change in overall richness, but a clear compositional shift	- Increased fecal acetate, propionate, and butyrate - Increased phospholipids - Lower plasma levels of l-carnitine and TMAO	- Lower TMAO and l-carnitine linked to reduced cardiovascular aging risk - Higher SCFAs suggest improved gut barrier function and reduced inflammation in aging - Favorable lipid profile (lower oxidized LDL-C and total cholesterol) supports vascular health and longevity	[29]
	- Increased *Faecalibacterium prausnitzii*, *Bacteroides fragilis*, and *Clostridium* cluster XIVa and IV - Decreased *Enterobacteriaceae* (including potential pathogens, like *Escherichia coli*) - Higher abundance of *Prevotella*, *Bacteroides*, and *Subdoligranulum* in long-term vegetarians	- Lower lipocalin-2 and small-chain fatty acid concentration after vegetarian intervention (short-term) - Higher genes for nitrogen assimilation due to lower amino acid intake	- Decreased *Enterobacteriaceae*, resulting in a lower risk of gut-derived endotoxemia and inflammaging - Increased *F. prausnitzii* and *Subdoligranulum* resultingbetter immune resilience in aging	[30]
	- Higher *Bacteroides*/*Prevotella* ratio and greater abundance of *Subdoligranulum*	- Lower fecal bile acid concentrations - Reduced lipocalin-2, a marker of gut inflammation	- Reduced bile acids and endotoxins encourage potentially lower cancer and metabolic aging risks	[31]

**Table 2:** Impact of different dietary nutrients on gut microbiota and gut health.

Basic Nutrient	Intervention Description	Test Model	Treatment Conditions	Outcome	Reference
Dietary fat	A high-fat diet comprising 72% fat (from corn oil and lard), 28% protein, 1% carbohydrate	Male C57bl6/J mice and ob/ob mice (C57bl6 background)	High-fat diet (72% fat) and/or antibiotic treatment (1 g/L ampicillin + 0.5 g/L neomycin in drinking water), n = 13–17 depending on group. Duration: 4 weeks. Location: Université Catholique de Louvain, Belgium, and Rangueil Institute of Molecular Medicine, Toulouse, France	High-fat diet altered gut microbiota, increased intestinal permeability, and elevated plasma lipopolysaccharides (metabolic endotoxemia), leading to inflammation, oxidative stress, adipocyte hypertrophy, insulin resistance, and glucose intolerance	[48]
	Total groups: control diet (CD), high-fat diet (HFD), HFD + oleic acid-derived compound (HFD-S1), HFD + omega-3 fatty acids (HFD-S2)	Female ICR (CD-1) outbred mice, 8 weeks old at study	HFD-S1: 1500 mg/kg/day oleic acid-derived compound HFD-S2: 3000 mg/kg/day EPA + DHA (omega-3 fatty acids) n = 6–8 mice per cage, 3 cages per group Duration: 8-week HFD induction followed by 7 weeks of supplementation Location: Institute of Food Science, Technology, and Nutrition (ICTAN-CSIC), Madrid, Spain	HFD alone increased gut dysbiosis (increased Firmicutes, Enterobacteriales; reduced *Bacteroidetes*, *Bifidobacterium* spp.) HFD-S1 (oleic acid compound) restored *Bacteroidetes* and Bifidobacterium levels and decreased clostridial cluster XIVa HFD-S2 (omega-3) increased *Lactobacillus* but had no effect on weight or other microbiota alterations	[49]
	Grouped as LF, low-fat diet; DIO-R, diet-induced obesity resistant; DIO-P, diet-induced obesity-prone diets	Male Sprague Dawley rats (initial weight ~262 g)	LF diet: 70% carbohydrate, 20% protein, 10% fat (SAT 25.1%, MUFA 34.7%, PUFA 40.2%), 3.85 kcal/g HF diet: 35% carbohydrate, 20% protein, 45% fat (SAT 36.3%, MUFA 45.3%, PUFA 18.5%), 4.73 kcal/g Duration: 8 or 12 weeks Location: University of California, Davis, CA, USA	Elevated gut inflammation markers in DIO-P Increased intestinal permeability and plasma lipopolysaccharides in DIO-P HF diet altered gut microbiota in all groups (increased Bacteroidales and Clostridiales; reduced total bacterial load), but only DIO-P showed increased Enterobacteriales	[50]
Dietary fiber	Randomized, crossover clinical trial Mixed fiber supplement from Revilife (Nantong Richen Bioengineering Co., Ltd., Nantong City, China)	12 healthy young adults (6 males, 6 females) aged 22–32 years	20 g/day mixed dietary fiber (polyglucan, inulin, resistant malt dextrin) for 4 days 4-day washout Total study duration: 16 days (including washout and baseline) Location: Xinjiang Medical University, Urumqi, China	Fiber intervention increased the abundance of *Alloprevotella*, *Parabacteroides*, and *Parasutterella* Decreased the abundance of *Adlercreutzia*, *Anaerovorax*, *Enterococcus*, *Intestinibacter*, and *Ruminococcus*2	[51]
	Meals are lyophilized and pre-packaged to control fiber intake; fiber source unspecified	Human participants: 19 healthy young adults (9 males, 10 females) aged 19–25 years	10 g/day or 40 g/day dietary fiber for 5 days, separated by 2-week washout periods Crossed over after the first phase (10 participants: 10 g → 40 g; 9 participants: 40 g → 10 g) All meals prepared and standardized (same ingredients, same soluble/insoluble fiber ratio) Total duration: 6 weeks Location: Grenoble University Hospital and INRA, Jouy-en-Josas, France	Increased *Prevotella*, *Coprococcus*, *Dorea* species and elevated short-chain fatty acids, like caproate and valerate Low richness participants showed more variable microbiota shifts with fiber intervention	[52]
Carbohydrates	Carbohydrate (in the form of oligofructose-enriched inulin). All were on a gluten-free diet (GFD) for at least 6 months prior to the trial	Human participants: 34 pediatric celiac disease patients (62% female), mean age 10 years	Oligofructose-enriched inulin (Synergy 1) (10 g/day), n = 18, and placebo (maltodextrin; 7 g/day), n = 16, as reference/clinical trials (34 pediatric celiac disease patients, 62% females, on a gluten-free diet) Synergy 1 (Orafti^®^) and placebo supplements were administered orally once daily Duration: 3 months. Location: University of Warmia and Mazury, Olsztyn, Poland	Significant increase in *Bifidobacterium* count in Synergy 1 group; stable *Clostridium leptum* count vs. decline in placebo; reduced *Lactobacillus* in both groups Metabolites: Synergy 1 group showed increased fecal acetate and butyrate, with total SCFAs rising by 31% from baseline	[53]
	LC (low-carbohydrate) group (n = 11) LC + HIIT: high-intensity interval training (LC-HIIT, n = 13): 10 sprints of 6 s with 9 s rests LC + MICT: moderate-intensity continuous training (LC-MICT, n = 12): 30 min cycling at 50–60% VO_2_ peak	50 overweight/obese young Chinese females, age 22.2 ± 3.3 years, BMI 25.1 ± 3.1 kg/m^2^	Low-carbohydrate diet (9% carbs, 23% protein, 68% fat of daily energy) maintained for 4 weeks, total daily intake ~1900 kcal LC-HIIT: 20 sessions over 4 weeks (2.5 min/session, sprint cycling) LC-MICT: 30 min continuous cycling at increasing intensity over 4 weeks. Location: University of Macau, China (Participants kept stable daily energy intake; food diaries monitored)	LC increase *Phascolarctobacterium* LC-HIIT decreased *Bifidobacterium* Both LC-HIIT and LC-MICT increased *Blautia* and reduced *Alistipes* (linked to type 2 diabetes)Changes in genera, like *Sutterella* and *Enterobacter*, correlated with body composition metrics Blood pressure changes associated positively with *Ruminococcus*, *Eubacterium*, *Roseburia* and negatively with *Faecalibacterium*, *Bacteroides*, and *Parabacteroides*	[54]
Protein	Mice were weaned directly onto the synthetic diets; a subset received the diet in the parental generation to assess long-term effects	Male BALB/c and RAG2 knockout mice and germ-free mice	aCD (animal protein control): 176 g/kg casein aHPD (animal high protein): 514 g/kg casein pCD (plant protein control): 173 g/kg wheat gluten pHPD (plant high protein): 500 g/kg wheat gluten DSS-induced colitis: 3% dextran sulfate sodium Chronic colitis: 3 cycles of DSS (5 days DSS + 9 days water each) Duration: 3 weeks of diet pre-treatment + acute/chronic colitis phases Location: Institute of Microbiology of the CAS, Prague, Czech Republic	aHPD-fed mice showed increased severity of both acute and chronic DSS colitis, altered microbiota: increased *Escherichia*, *Enterococcus*, *Streptococcus*, reduced *Lactobacillus*, *Bifidobacterium*, and increased abundance of *Candida tropicalis* aHPD mice had reduced bacterial alpha diversity and shifted functional microbial genes (reduced barrier function pathways, increased motility/secretion)	[55]
	P14: 14% protein diet P30: 30% protein diet P53: 53% protein diet	Male C57BL/6 mice (n = 132 DSS-treated, plus 12 healthy controls)	Isocaloric diets differing only in protein content (milk protein: casein + whey) Treatment started on day 7 (post-inflammation peak) and continued for 3, 6, or 21 days Food and water provided ad libitum Outcomes assessed on days 10, 13, and 28 Location: AgroParisTech and INRA labs, Palaiseau, France	P30 diet improved mucosal healing, reduced intestinal permeability, increased epithelial proliferation P30-fed mice showed increased colonization by butyrate-producing bacteria during resolution phase	[56]
		Female C57BL/6J mice	REF: low-fat control diet HFS: high-fat diet (25%) with high sucrose (43%) HFP: high-fat diet (25%) with high protein (43%) Ad libitum feeding from 3 weeks of age until study endpoints (up to ~95 weeks) REF (9% fat, 33% sucrose, 33% protein); HFS (25% fat, 43% sucrose); HFP (25% fat, 43% protein)	Gut microbiota changed significantly with age, especially at 16 months, and aging is linked to decreased firmicutes to *Bacteroidetes* ratio (in REF and HFP groups) Phylotypes driving age-related shifts included *Akkermansia muciniphila*, *Sphingomonas*, *Desulfovibrio*, and *Olsenella* Gut microbial changes with age were more prominent than changes due to the protein/sucrose ratio. Survival was lower in HFS mice; HFP mice maintained better longevity, metabolic profile, and microbial diversity—bacterial diversity declined with age across all diet groups	[57]
Vitamin	Vitamin A	306 human infants	50,000 IU vitamin A or placebo, orally within 48 h of birth—no additional interventions during early infancy; follow-up through 15 weeks and again at 2 years—location: Dhaka, Bangladesh	At 2 years, plasma retinol was positively associated with *Actinobacteria* (especially *Bifidobacterium*) and *Akkermansia* in girls Vitamin A supplementation increased *Bifidobacterium* abundance in boys during early infancy but not in girls Gut microbiota diversity changed with age, and supplementation had sex-specific effects persisting into toddlerhood	[58]
	Vitamin D	25 human participants with low vitamin D levels (25(OH)D < 50 nmol/L): 8 with active ulcerative colitis (UC) 9 with inactive UC 8 non-IBD controls	40,000 IU vitamin D_3_ (cholecalciferol) per week for 8 weeks Total dose: 320,000 IU over study period Participants assessed pre- and post-intervention for inflammatory markers and gut microbiota composition Location: Harrow, UK	Increase in Enterobacteriaceae abundance after supplementation. No significant shifts in *Ruminococcus gnavus*, *Akkermansia*, *Bifidobacteria*, or SCFA-producing Clostridia. Trends suggested reduced *R. gnavus* after treatment, but not statistically significant	[59]
Minerals	Phosphorus	62 healthy adult participants (30 men, 32 women), mean age 29 ± 7 years, mean BMI 24 ± 3 kg/m^2^	2-week placebo run-in for all groups 3 intervention arms for 8 weeks: P1000/Ca0: 1000 mg phosphorus/day P1000/Ca500: 1000 mg phosphorus + 500 mg calcium/day P1000/Ca1000: 1000 mg phosphorus + 1000 mg calcium/day Phosphorus as monosodium phosphate; calcium as calcium carbonate Intake via sherbet powder diluted in water, twice daily	P1000/Ca1000 group (men only) showed a significantly altered gut microbial community compared to Ca0 and Ca500 *Clostridium* XVIII was more abundant in men in the Ca1000 group No significant changes in microbial diversity in women	[60]
	Iodine	Female ICR mice (3 weeks old), n = 6 per group Groups: control (standard diet) control + iodine (KIO_3_) high-fat diet (HFD) HFD + iodine (KIO_3_)	18 μg/kg/day potassium iodate (KIO_3_) via daily oral gavage HFD: 34.9% fat, 5.21 kcal/g; control diet: 4.62% fat, 3.45 kcal/g. Duration: 8 weeks. Iodine supplementation following HFD induction	Iodine increased pathogenic bacteria in obese mice (e.g., *Clostridium*, *Enterococcus*, *Fusobacterium nucleatum*) and reduced probiotics (*Lactobacillus*, *Bifidobacterium*, *F. prausnitzii*) Iodine had opposite effects in normal mice, increasing beneficial microbes and lowering inflammatory bacteria	[61]
Polyphenols	Anthocyanins (blackberry anthocyanin-rich extract/BE)	Male Wistar rats, n = 24 total Groups: control diet (C) control + BE (C + BE) high-fat diet (HF) high-fat diet + BE (HF + BE)	BE: 25 mg/kg/day, delivered in food pellets Duration: 17 weeks total Diets: standard or high-fat diet (60% calories from fat)	BE altered gut microbiota and attenuated neuroinflammation, a hallmark of cognitive aging BE restored microbial diversity disrupted by HF diet, increased *Pseudoflavonifractor* and *Oscillobacter*, and reduced pro-inflammatory bacteria (e.g., *Ruminococcus*). BE also reduced fecal LPS (lipopolysaccharide), hinting at better gut barrier function	[62]

**Table 3:** Differences between the Mediterranean, DASH, and MIND diets.

Feature	Mediterranean Diet (MD)	DASH Diet	MIND Diet
Overall Aim	Promote longevity and reduce chronic disease risk through balanced, traditional dietary patterns	Prevent and control hypertension while supporting cardiovascular health	Protect brain health and reduce the risk of dementia/Alzheimer’s disease
Core Principles	Plant-forward diet, daily olive oil, moderate fish/poultry, limited red meat, moderate wine	High intake of fruits, vegetables, whole grains, low-fat dairy, lean protein, reduced sodium	Hybrid of DASH and MD with targeted emphasis on brain-protective foods
Primary Food Sources	Vegetables, fruits, legumes, nuts, whole grains, olive oil, fish, moderate dairy, and wine	Fruits, vegetables, whole grains, low-fat dairy, nuts, seeds, poultry, fish	Leafy greens, berries, nuts, whole grains, olive oil, beans, fish, poultry
Restricted Foods	Red/processed meat, refined grains, added sugars, butter, cream	High-sodium foods, red/processed meat, sweets, sugar-sweetened drinks	Red meat, butter/margarine, cheese, pastries, fried/fast food
Key Nutrients	Monounsaturated fats, fiber, antioxidants, polyphenols, omega-3s	Potassium, calcium, magnesium, fiber, lean proteins, low sodium	Vitamin E, folate, omega-3s, antioxidants (especially from berries and greens)
Lifestyle Factors	Encourages communal eating, seasonal/local foods, physical activity	Portion control, sodium restriction, balanced nutrient intake	Focus on consistent intake of neuroprotective foods rather than calorie restriction
Evidence-Based Health Outcomes	Lower risk of cardiovascular disease, diabetes, cancer; improved longevity	Clinically proven to lower blood pressure and cardiovascular risk	Slower cognitive decline, reduced risk of Alzheimer’s and neurodegeneration

**Table 4:** Effects of the DASH, MIND, and Mediterranean diets on healthy aging.

Diet type	Study Details	Effect on Aging	References
DASH diet	147 participants, age ≥60, diagnosed with hypertension and/or hyperlipidemia, 12-month duration, randomized into MNT* vs. information-only group, 3 MNT sessions over one year, dietary recalls and knowledge assessments at baseline, 6 months, and 12 months, DASH adherence measured via nutrient scoring, study type: interventional, randomized controlled trial	↓ blood pressure → ↓ stroke risk by 20–40%, ↓ LDL cholesterol by 1–20.9% → ↓ atherosclerosis progression, ↑ potassium intake → ↓ salt sensitivity, especially in older adults, ↓ saturated fat intake → ↓ cognitive decline risk by 15%, ↑ fiber intake → ↓ inflammation and improved vascular aging, ↓ sodium intake → ↓ arterial stiffness and improved brain perfusion, ↑ DASH adherence → ↑ cardiovascular resilience in aging	[133]
	Older adults aged ≥60, studies span 6 months to 6 years, DASH diet assessed through food frequency questionnaires and nutrient scoring, cognitive outcomes measured using MMSE (mini-mental state examination), CASL (comprehensive assessment of spoken language), and memory tests	↑ MMSE scores by 1.3–2.1 points in high adherence groups, ↓ risk of cognitive decline by 11–25% over 4–6 years, ↑ verbal memory performance by 15% with combined DASH and exercise, better vascular aging, ↓ risk of dementia by 20% in long-term adherence	[134]
	4169 participants aged 45 to 84, multi-ethnic sample including White, African American, Hispanic, and Chinese American adults, free of cardiovascular disease at baseline, cognitive assessments conducted in 2011–2012 and 2016–2018, cognitive tests included digit symbol coding, cognitive abilities screening instrument, and digit span, study type was a prospective cohort	Improved processing speed, with digit symbol coding score rising by 1.3 points per 1 sd increase in DASH adherence, global cognition also improved, with cognitive abilities screening score increasing by 0.9 points per 1 sd, working memory showed no change, cognitive decline over five years was slower in participants with higher DASH adherence, aging effects were more pronounced in White and Chinese American groups	[135]
MIND diet	4066 participants, age ≥55, Chinese adults, median follow-up 3 years, cognitive tests conducted in 1997, 2000, 2004, and 2006, MIND diet score range 0–12, dietary intake assessed via 3-day 24 h recall and household weighing, cognitive function measured using telephone interview for cognitive status-modified, study type: observational, prospective cohort study, meta-analysis included 8 studies with 26,103 participants from China, the United States, and Spain	↑ global cognitive z-score by 0.110 per 3-point increase in MIND score, ↑ verbal memory score by 0.102 per 3-point increase, ↑ cognitive function by 0.042 per sd increase in meta-analysis, ↓ annual cognitive decline by 0.010 units per sd increase, ↑ cognition equivalent to being 1 year younger per 3-point increase	[136]
	207 participants, age 34.1 ± 6.0 years, middle-aged adults, east-central Illinois, USA, cross-sectional study, data collected 2015–2020, MIND diet score range 3.0–12.5, average adherence ~49%, dietary intake assessed via dietary history questionnaire II (DHQII)	↑ cognitive processing speed during incongruent trials, faster neural efficiency	[137]
	604 participants, age ≥65, cognitively unimpaired, overweight (BMI ≥25), family history of dementia, suboptimal diet (MIND score ≤8), recruited from Chicago and Boston, randomized 1:1 to MIND diet vs. control diet (both with mild caloric restriction), intervention duration 3 years, dietary counseling provided to both groups, cognitive function assessed via 12-test battery (converted to z-scores), brain imaging (MRI) conducted in nonrandom subsample (n = 200), study type: two-site randomized controlled trial	↑ global cognition score by 0.205 standardized units in MIND group vs. 0.170 in control group, mean difference = 0.035 (95% CI: −0.022 to 0.092, *p* = 0.23), no significant difference in cognitive domain scores, ↑ MIND diet score by 3.3 points in MIND group vs. 0.7 in control	[138]
Mediterranean diet (MD)	105 participants, age ≥60, at risk of undernutrition and cognitive decline, 6-month duration, 3 groups (diet + exercise, diet only, control), protein target 1.5 g/kg/day, energy 30 kcal/kg/day, exercise 2×/week 30–60 min, key foods delivered 3 months, personalized counseling, study type: interventional, randomized controlled trial	MNA score ↑ by 3.2 points, cognitive score ↑ by 11%, muscle mass ↑ by 8%, physical function ↑ by 9%, appetite score ↑ by 15%, inflammation markers ↓ by 12%	[139]
	Large cohort studies (NHS, HPFS), follow-up duration >30 years, dietary patterns analyzed include Mediterranean, DASH, plant-based, Nordic, Okinawa, study type: observational, includes cohort and case–control studies	Lean body shape linked to 17% higher chance of healthy aging, replacing 5% saturated fat with polyunsaturated fat ↓ mortality risk by 15–27%, high nut intake ↓ death rate by 20%, high olive oil intake ↓ CVD risk by 16%, high anthocyanin intake ↓ cognitive decline odds by 24%	[140]
	15 studies, age range mostly ≥60, sample sizes varied (30–1000+), duration ranged from 6 months to 4 years, diets assessed were Mediterranean, Ketogenic, MIND, cognitive decline or dementia as primary outcome, interventions included dietary counseling, food provision, or self-reported adherence, study type: observational and interventional, includes cohort studies, randomized controlled trials	Improved global cognition scores, ↓ Alzheimer’s disease risk by 53% with high adherence, ↑ memory retention and executive function, ↓ neuroinflammation markers, ↑ brain-derived neurotrophic factor (BDNF) levels, ↑ hippocampal volume in long-term adherence, ↓ cognitive decline rate over 4.5 years, ↑ antioxidant intake → better neuronal protection	[141]
	604 participants, age 65–84, at risk for Alzheimer’s disease, 3-year duration, personalized dietary counseling, food provision included, cognitive assessments every 6 months, study type: interventional, randomized controlled trial	↑ global cognition scores by 35% in high adherence group, ↓ Alzheimer’s disease risk by 53% with consistent adherence, ↑ memory and executive function by 20–25% over 3 years, ↓ oxidative stress markers by 30%, ↑ brain-derived neurotrophic factor (BDNF) levels by 25%, ↓ cognitive decline rate by 30% over 4.5 years, ↑ antioxidants	[142]

* Medical nutrition therapy (MNT) is a personalized nutrition intervention, provided by a dietitian or nutrition professional, aimed at managing a specific nutrition-related condition. The treatment is based on a comprehensive nutrition assessment. The upward arrow indicates the increments, the downward arrows indicate reductions and the right arrows indicate change.

## Abbreviations

The following abbreviations are used in this manuscript:

SCFA	Short-Chain Fatty Acid
MD	Mediterranean Diet
PUFA	Polyunsaturated Fatty Acid
MUFA	Monounsaturated Fatty Acid
TMA	Trimethylamine
TMAO	Trimethylamine-N-oxide
LPS	Lipopolysaccharide
MMKD	Modified Mediterranean Ketogenic Diet
BMI	Body Mass Index
DNA	Deoxyribonucleic Acid
ROS	Reactive Oxygen Species
NO	Nitric Oxide
8-OHdG	8-hydroxy-2′-deoxyguanosine
CALERIE	Comprehensive Assessment of Long-term Effects of Reducing Intake of Energy
DASH	Dietary Approaches to Stop Hypertension
MIND	Mediterranean-DASH Intervention for Neurodegenerative Delay
ITF	Inulin-Type Fructan
GOS	Galactooligosaccharide
AP	Aloe Polysaccharide
FXR	Farnesoid X Receptor
TGR5	Takeda G Protein-Coupled Receptor 5
IgA	Immunoglobulin A
TJP2	Tight Junction Protein 2
HDAC	Histone Deacetylase
BDNF	Brain-Derived Neurotrophic Factor
AMPK	AMP-Activated Protein Kinase
mTOR	Mechanistic Target of Rapamycin
MACs	Microbiota-Accessible Carbohydrates
UNESCO	United Nations Educational, Scientific, and Cultural Organization
